# Reconstructing Mayaro virus circulation in French Guiana shows frequent spillovers

**DOI:** 10.1038/s41467-020-16516-x

**Published:** 2020-06-05

**Authors:** Nathanaël Hozé, Henrik Salje, Dominique Rousset, Camille Fritzell, Jessica Vanhomwegen, Sarah Bailly, Matthieu Najm, Antoine Enfissi, Jean-Claude Manuguerra, Claude Flamand, Simon Cauchemez

**Affiliations:** 1 Mathematical Modelling of Infectious Diseases Unit, Institut Pasteur, UMR2000, CNRS, 75015 Paris, France; 2 Department of Genetics, University of Cambridge, Cambridge, UK; 3 Arbovirus National Reference Center, Institut Pasteur in French Guiana, Cayenne, French Guiana; 4 Epidemiology Unit, Institut Pasteur in French Guiana, Cayenne, French Guiana; 5 Environment and Infectious Risks Unit, Institut Pasteur, Paris, France

**Keywords:** Statistical methods, Viral infection, Epidemiology

## Abstract

Characterizing the circulation of Mayaro virus (MAYV), an emerging arbovirus threat, is essential for risk assessment but challenging due to cross-reactivity with other alphaviruses such as chikungunya virus (CHIKV). Here, we develop an analytical framework to jointly assess MAYV epidemiology and the extent of cross-reactivity with CHIKV from serological data collected throughout French Guiana (N = 2697). We find strong evidence of an important sylvatic cycle for MAYV with most infections occurring near the natural reservoir in rural areas and in individuals more likely to go to the forest (i.e., adult males) and with seroprevalences of up to 18% in some areas. These findings highlight the need to strengthen MAYV surveillance in the region and showcase how modeling can improve interpretation of cross-reacting assays.

## Abstract (web summary)

Mayaro virus (MAYV) is an emerging arbovirus, but cross-reactivity with other alphaviruses makes analysis of its epidemiology difficult. Here, the authors develop an analytical framework to assess MAYV epidemiology and find evidence for an important sylvatic cycle and seroprevalences of up to 18% in some areas of French Guiana.

## Introduction

Arboviruses constitute an important and evolving threat for public health. Mayaro virus (MAYV), an alphavirus often cited as a likely candidate for the next major arbovirus emergence^1–4^, is mainly transmitted by forest-dwelling Haemagogus mosquitoes to nonhuman primates and other mammalian reservoirs and results in symptoms similar to those of dengue or chikungunya^5^. Practices such as deforestation and human activities in forested regions^6^ increase the risk that MAYV will move from a sylvatic cycle (i.e., circulates in an animal reservoir but with only sporadic human infections) to a domestic cycle (i.e., self-sustaining transmission in humans) with important consequences for public health. Active circulation has been confirmed in various areas including urban settings in Caribbean area, South and Central America^7–10^, but surprisingly little is known about its circulation in human populations.

Seroprevalence studies that quantify the proportion of the population with antibodies against MAYV can help address such knowledge gap in the level of circulation and spatial extent^11^ and support risk assessment of this emerging pathogen. However, following the widescale circulation of CHIKV in the Americas since 2013, the evaluation of MAYV infection is greatly complicated by serological cross-reactivity where CHIKV infected individuals may experience a rise in MAYV antibody measures, even if they have not been infected by MAYV. This problem of cross-reactivity hampers serological studies across many different pathogens^12^.

Here, we demonstrate that by jointly analyzing serological results for both MAYV and CHIKV alongside data on the age and location of participants, we can simultaneously reconstruct the history of circulation of the viruses and the extent of cross-reactivity. We apply our approach to MAYV and CHIKV in French Guiana, a territory that has seen a CHIKV epidemic between February 2014 and October 2015^13–15^, as well as documented MAYV cases^16,17^. We randomly collected 2697 population-representative serum samples from all age-groups from throughout French Guiana^18^ (Methods and Supplementary Table 1). For each serum sample, we measured antibody responses to both MAYV and CHIKV using a multiplexed microsphere-based IgG immunoassay (MIA) that returned a relative fluorescence intensity (RFI) for each pathogen^19^ (see Methods section).

## Results

### Serology of MAYV and CHIKV

**Fig. 1:**
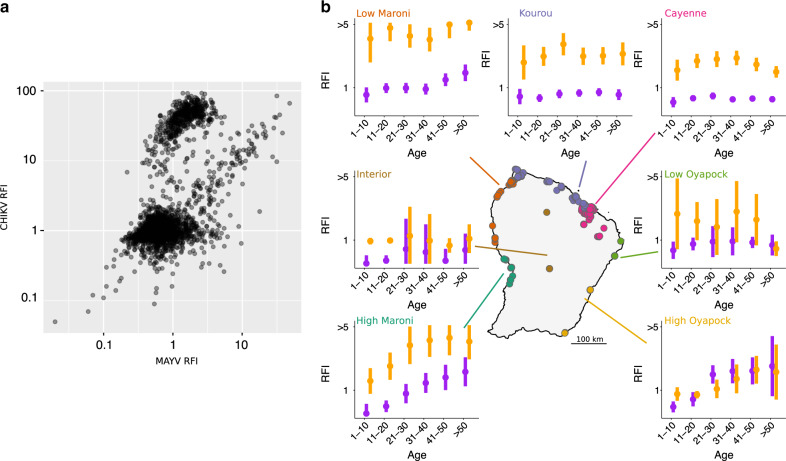
Serology of MAYV and CHIKV in French Guiana. **a** MAYV and CHIKV relative fluorescence intensity (RFI) for the 2697 individuals included in the study. **b** Map of French Guiana showing the location of samplings in 22 municipalities. Colors in the map indicate the seven geographical clusters. Mean +/− SEM of the RFI stratified in 10-year age groups is shown for each region for MAYV (purple) and CHIKV (orange). Sample sizes for each age class are given in Supplementary Table 4.

We find that almost all participants with a MAYV RFI signal also exhibit a signal for CHIKV (Fig. 1a). In the absence of a good understanding of the antibody response following exposure and of cross-reactivity, we cannot reliably estimate the proportion of participants historically infected by MAYV from such laboratory results alone. If an increase in MAYV RFI was solely due to cross-reactivity with CHIKV, MAYV RFI should be roughly proportional to CHIKV RFI in each region of French Guiana. However, we observe important differences between regions (Fig. 1b). For example, while the average MAYV RFI is much lower than the average CHIKV RFI in Cayenne, it is higher in High Oyapock among participants aged >20 years old (Fig. 1b). We hypothesize that the true level of circulation of each virus and the level of cross-reactivity can be identified if we integrate in our analysis the fact that individuals with shared characteristics—age, region, sex, housing, and income—tend to have correlated risks of historic infection. Our approach jointly estimates the location-specific annual force of infection (FOI, per capita rate at which a susceptible individual gets infected) for MAYV and CHIKV modulated by pathogen-specific socio-demographic factors, and how RFIs change to both the infecting and non-infecting virus following infection (see Methods). In a simulation study, we find that our approach is able to reliably estimate model parameters and reconstruct viral circulation histories (See Supplementary Tables 2 and 3).

Using this integrative analytical framework, we estimate that MAYV RFI increases on the log scale by 2.2 (95% credible interval (CrI): 2.1 –2.3) for an individual historically infected with MAYV and by 0.22 (95% CrI: 0.20–0.23) after a CHIKV infection while CHIKV RFI increases by 1.05 (95% CrI: 1.01–1.1) after a MAYV infection and by 3.64 (95% CrI: 3.60–3.67) after a CHIKV infection (Tab S4).

### Assessing the history of virus circulation

**Fig. 2:**
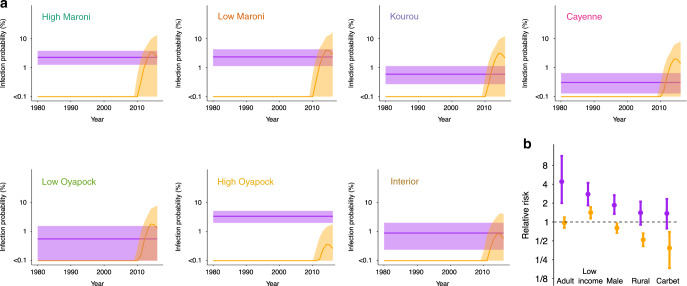
Model estimates of the determinants of infection. **a** Best model fit of the yearly probability of infection by MAYV (purple) and CHIKV (orange) of an adult male in the different regions of French Guiana. Solid lines indicate the mean probability of infection and envelopes the 95% credible intervals. **b** Relative risk of MAYV (purple) and CHIKV (orange) infection of individuals above 20 years old (relative to younger individuals), low income (relative to high income), males (relative to females), living in a rural area (relative to an urban area), and living in a carbet (jungle housing) relative to other types of habitations. Data are presented as the mean posterior with error bars denoting 95% credible intervals and the dashed line represents a relative risk of 1.

In addition to cross-reactivity, our model assesses the history of circulation and infection risk factors for each virus. For MAYV, our best fitting model (see Methods section Alternative models of virus circulation and Supplementary Tables 6 and 7) assumes that the force of infection for MAYV has remained stable over time, with important regional variations (Fig. 2a). At any point in time, we find that males are 1.9 (95% CrI: 1.3–2.7) times as likely to be infected by MAYV than females, and adults are 5.1 (95% CrI: 2.2–10.5) times as likely to be infected than children (Fig. 2b). Living in a carbet (typical Native American cabin without walls) is a risk factor for MAYV infection (OR: 1.4, 95% CrI: 0.8–2.3). These estimates support the scenario of a sylvatic transmission cycle for MAYV where most infections occur near the natural reservoir in rural areas and where individuals more likely to go to the forest (i.e., adult males) are also at higher risk of infection.

For CHIKV, our best fitting model (see Methods section Alternative models of virus circulation) correctly identifies that the virus emerged only recently in French Guiana (Fig. 2a)^20^ and predominantly circulated in the coastal and urbanized areas, as well as the north western region of the territory. Contrasting with MAYV, we find that females are more likely to be infected by CHIKV than males (relative FOI is 1.2, 95% CrI: 1.0–1.4) and children are as likely as adults to be infected (Fig. 2b). We find an increased risk of CHIKV infection for individuals that spend more time at home. We assessed whether this observation could help explain the increased level of CHIKV infection among women. In the survey, participants were asked the average time they spend at home per day. We found that females were 1.38 (95% CrI: 1.13–1.69) times more likely than men to spend >16 h per day at home. Spending time at home was also associated with infection by CHIKV (OR, 95% CrI: 1.84 (1.41–2.40)), but not significantly associated with MAYV infection (OR, 95% CrI: 1.30 (0.79–2.14)). These findings are consistent with a previous study on CHIKV transmission in Bangladesh, which suggested that the increased time women spend within and around their home compared to men was responsible for an increased risk of infection^21^. We also tested whether the differences between males and females could be explained by differential boosting of the RFI rather than different viral exposure levels. We ran the same model using only males and only females. We found that the overall prevalence was not modified by this additional assumption, although we found a slightly larger mean boosting for females, but larger individual variations for males (Supplementary Table 8). There is a good adequacy between model predictions and the data (Supplementary Figs. 1 and 2).

### Model-based classification of infection

**Fig. 3:**
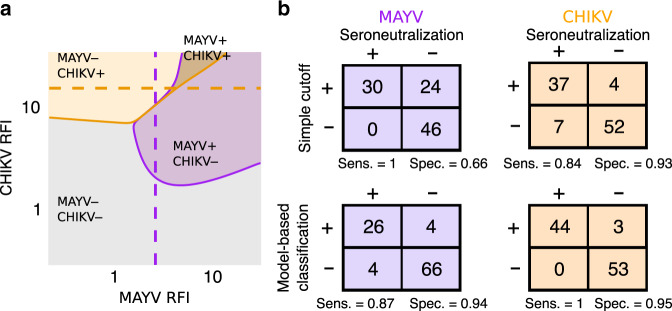
Classification of MAYV and CHIKV infections. **a** Graphical representation of the model-based classification that determines the infection status for MAYV and CHIKV from the measured MAYV and CHIKV RFIs. For a classification with a simple cut-off, MAYV positive samples would lie on the right of the dashed purple curve, and CHIKV positive samples above the orange curve. The boundaries were plotted considering a probability of MAYV infection of 5% and CHIKV infection of 20% (see Supplementary Fig. 4 for a sensitivity analysis where the infection probabilities are changed). **b** Contingency tables comparing the simple cutoff and the model-based classifications using the seroneutralization assay (performed on 100 individuals) as a reference.

Once parameters characterizing cross-reactivity and the force of infection have been estimated, the model can be used to improve the interpretation of serological assays and estimate the prevalence of infection. We propose a model-based classification that derives for each possible value of the assays the probability of infection by MAYV and/or CHIKV and classifies the result as infected if the estimated probability of infection is above 50% (Fig. 3a). To validate the model-based classification, 100 sera were selected for further testing with anti-MAYV and anti-CHIKV microneutralization tests (MNTs). Using the results from the MNTs as the gold standard, we find that the model-based classification that uses both RFI values performs substantially better than a classification based on a simple optimized cut-off (Fig. 3b and Supplementary Figs. 3 to 5).

### Geographic distribution of seroprevalence

**Fig. 4:**
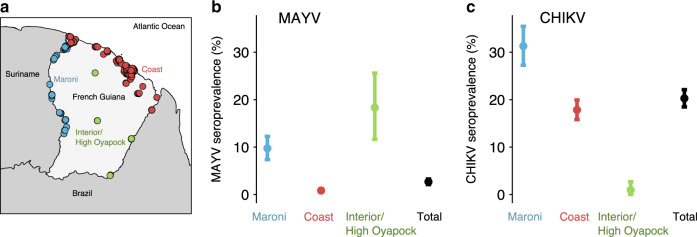
Geographic distribution of MAYV and CHIKV seroprevalence. **a** Three regions of French Guiana with distinct epidemiological characteristics are derived using the model estimates. **b**, MAYV and **c**, CHIKV seroprevalence estimated using the model-based classification (Maroni, *n* = 618; Coast, *n* = 1919, Interior, *n* = 160, Total, *n* = 2697). Points and error bars indicate the mean and 95% confidence intervals of the weighted seroprevalence, respectively.

From the analysis of transmission dynamics in the seven regions (Fig. 1b), we identified three larger regions (Maroni, Coast, high Oyapock/Interior) that allowed us to divide the country into areas with distinct epidemiological characteristics (Fig. 4a). The model-based classification was applied to data from each of these regions with survey weights to derive representative estimates of seroprevalence (Fig. 4b, c). We estimate that 20.3% (95% confidence interval (CI): 18.5–22.1%) of the population was infected by CHIKV, predominantly in the urban areas of the Coast and along the Maroni river (Surinamese border) (Supplementary Fig. 6, Supplementary Tables 9 and 10). This geographical distribution of CHIKV appears similar to that previously found for Zika virus (ZIKV)^18^. For both ZIKV and CHIKV, risk may be driven by the repartition of Aedes aegypti, which may have reached almost all inhabited areas but not the most remote villages. To date no study has reported the presence of *Ae.aegypti* populations in the most remote villages including Antecume Pata, Trois-Sauts and Camopi villages^22^ where CHIKV seroprevalence rates varied from 0% to 1.42%. Furthermore, we estimate that 2.8% (95% CI: 2.2–3.4%) of the population was infected by MAYV (number of seropositive individuals: 7263; 95% CI: 5764–8868 in a population of 258,000). Highest seroprevalences were observed in sparsely populated high Oyapock/Interior region (<1% of French Guiana population), in agreement with previous studies which found high MAYV seroprevalence in this region^17^. A majority of infections inferred by the model (68%) came from the Maroni region (Supplementary Fig. 7) where 10.0% (95% CI: 7.6–12.6%) of the 49,169 inhabitants have been infected, and the annual probability of infection is 0.45% (95% CrI: 0.35–0.56) – or 223 (95% CrI: 175–271) new infections per year (Supplementary Table 11). These results show that MAYV transmission is higher in remote forested areas of French Guiana and are consistent with a sylvatic transmission of MAYV. We also found individuals with historical infections of MAYV in the urban regions characterized by low MAYV transmission. However, from our serological data, it is not possible to determine whether these infections occurred in these regions or during travel to other areas.

Had we inferred infections with a simple optimized cut-off, cross-reactivity with CHIKV would have led to overestimating the number of historical MAYV infections by almost 100% (estimated number of infections: 13,838 compared to 7263 with our model-based classification) and would have wrongly indicated a recent, urban outbreak (Methods section Analysis with a single cutoff). For MAYV, our best fitting model had a constant risk of infection over time, with the individual risk of infection increasing with age. As cross-sectional serological surveys do not allow us to distinguish between variations in age and in time, this result could be equally explained by larger rates of infection in the past and no difference in exposure between children and adults. However, there is evidence of ongoing MAYV transmission in French Guiana^16^, and MAYV is transmitted by the forest-dwelling mosquito Haemagogus, which is consistent with exposures in adult men who work in the forest.

To assess the risk of future MAYV emergence, it is important to understand how the CHIKV outbreak affected the susceptibility to MAYV. With our model-based classification, we found only five individuals with evidence of historical infections by both viruses. While this may be indicative of cross-immunity between the two alphaviruses, it may also be due to the vectors occupying different ecological niches. Serological assays alone are unable to differentiate between these potential mechanisms. Future animal challenge models may help in this context. Our results are specific to the E2 antigens used in the assay. Future studies that use different antigens would likely result in different estimates of cross-reactivity.

The epidemiological interpretation of serological surveys is often dogged by cross-reactivity between circulating pathogens^23^. Our study shows how modeling techniques integrating multiple data streams may overcome this issue, with important implications for the interpretation of pathogen serosurveys. Here, this allowed us to determine the level of circulation of MAYV, which is non-negligible in some parts of French Guiana. Our results highlight the need to strengthen surveillance for MAYV in the region so as to be able to quickly detect any substantial change in MAYV circulation patterns that may be indicative of a rise to emergence.

## Methods

### Study design and participants

A cross-sectional population-based serological survey and household interviews was conducted in French Guiana between June and October 2017. We reproduce here details on the random household selection, sampling weights, interviews, ethical considerations that were described in Flamand et al.^18^. The French Guianese territory is composed of 22 municipalities that we broke down into seven geographical areas for the statistical analysis. The areas and number of participants are given in Supplementary Table 1.

We estimated the sample size for this survey at 2500 persons distributed in the French Guiana territory based on a 50% seroprevalence, 95% confidence, 90% power and a cluster effect. To reach the desired sample size, a total of 1600 households were randomly selected for possible participation in the study from household databases maintained by the Geographic information and knowledge dissemination unit of the Regional environment, planning and housing agency and the National Institute of Economic and Statistical Information (INSEE). A stratified simple random sampling was adopted to select households allowing an over-representation of the isolated and small municipalities. The global sampling fraction of the households was 1:49 varying from 1:103 to 1:5 according to the municipality.

We employ the following notation to describe the study design:

– *i:* one of the 22 strata (municipalities);
– *M*_*i*_: number of primary sampling units (households) in the *i*th stratum, *i* = 1, …, 22;
– *S*_*i*_: number of primary sampling units (households) selected from the *i*th stratum, *i* = 1, …, 22;
– *m*_*i*_: number of primary sampling units (households) actually enrolled in the study from the *i*th stratum, i = 1, …, 22;
– *P*_*i*_*:* number of individuals living within the *i*th stratum, *i* = 1, …, 22 (census data);
– *p*_*i*_*:* number of individuals actually enrolled in the study from the *i*th stratum, *i* = 1, …, 22;

We considered that, in each municipality *i*, the probability of selecting a particular subject was equal to the probability of selecting his household and was (*m*_*i*_*/M*_*i*_), corresponding to a statistical weight equal to (1/*m*_*i*_*/M*_*i*_) = (*M*_*i*_*/m*_*i*_). This statistical weight indicates the number of people in the population represented by each subject in the sample.

We applied a post-stratification adjustment to each of these weights to arrive at the final statistical weight for each subject. This adjustment helped us to weight the age-sex groups within each municipality to match the distribution in the French Guiana total population. Ten age groups ([2–5 years] [5–10], [10–15], [15–20], [20–25], [25–35], [35–45], [45–55], [55–65], and ≥65 years) were defined within male and female groups, and for each age-sex subgroup, we applied an adjustment factor *c*_*ijk*_ to obtain a final statistical weight *w*_*ijk*_ = *(M*_*i*_*/m*_*i*_)*c_*ijk*_, where *i*, *j*, *k* are the indices of municipalities, sex groups, and age groups, respectively.

### Ethical considerations

Publicity and information about the survey was provided through the media and contact with local and national authorities. Fieldworker teams including investigators, nurses or medicine residents were trained to visit all households, explain the project objectives, and, when allowed, collect participant’s signatures in a free and informed consent form and carry out the interviews. All members of selected households who were 2–75 years of age were invited to take part in the study during a preliminary face-to-face interview. For all participants under 18 years of age, one or two responsible adults signed the informed consent. A specific educational-style comic book was designed for children 6 to 17 years of age to explain, in an understandable way, the nature and objectives of the survey and inform them about the voluntary nature of the participation of the study and their rights to access and rectify their personal information. The study was recorded on Clinicaltrials.gov (ID: NCT03210363) and approved by the “Sud-Ouest & Outre-Mer IV” Ethical Research Committee (No.CPP17-007a/2017-A00514-49) and by the French Data Protection Authority (No.DR-2017-324) responsible for ethical issues and protection of individual data collection.

### Blood sample collection

Blood samples were collected into 5 mL gold BD Vacutainer SST II advance tubes with gel for serum separation (Becton-Dickinson, USA). Immediately after puncture, samples were stored at 4 °C–8 °C until centrifugation within 12 h. Sera were then frozen and stored at −20 °C until use at the National Reference Center for arboviruses in Institut Pasteur in French Guiana.

### Multiplex microsphere-based immunoassay

Collected sera were tested using an in-house microsphere-based multiplex immunoassay (MIA) based on recombinant antigens. The recombinant ectodomain of the CHIKV and MAYV envelope E2 glycoproteins were used for the capture of specific IgG antibodies, whereas a recombinant human protein (O6-methylguanine DNA methyltransferase) was used as control antigen in the assay. Distinct MagPlex microsphere sets (Luminex Corp., Austin, TX) were, respectively, bound to viral and control proteins using the amine coupling kit (Bio-Rad Laboratories, Hercules, CA) according to manufacturers’ instructions. The MIA procedure was performed as described previously with minor modifications^24^. Briefly, microsphere mixtures were sequentially incubated in the dark under constant shaking with a 1:400 dilution of serum samples and 4 μg mL^−1^ anti-human IgG phycoerythrin-conjugated antibody (Jackson Immunoresearch, West Grove, PA). After the final incubation, the median fluorescence intensity (MFI) of each microsphere set was quantified using a MagPix instrument (Bio-Rad Laboratories). For each sample, the CHIKV and MAYV Relative Fluorescence Intensities (RFI) were calculated by dividing the MFI signal measured for the CHIKV or MAYV microsphere sets by the MFI signal obtained for the control microsphere set.

### Seroneutralization

To validate the model-based classification, 100 sera were selected for further testing with anti-MAYV and anti-CHIKV microneutralization tests (MNTs). Briefly MNTs were conducted in serial 2-fold dilutions of heat inactivated sera starting at 1:10 mixed in equal volume with 100 tissue culture infectious dose 50 (TCID 50) of MAYV or CHIKV (French Guiana strains). After incubation at 37 °C for 1 h, mixtures were transferred onto 96 well tissue culture plates containing subconfluent Vero cells. Plates were incubated at 37 °C for 6 days for MAYV and 5 days for CHIKV before lecture of cytopathic effects. The neutralization titer is expressed as the reciprocal of the highest serum dilution at which infection is blocked. A serum is considered positive for titer above 10. The individuals were selected as follows: 50 individuals from Cayenne (low MAYV seroprevalence) and 50 individuals from Maroni and Oyapock (high MAYV seroprevalence).

### Urbanization level

Urbanization level was obtained from a land use classification based on the proportion of households within a 1km-buffer (Rural: *p* < 50%; Urban: *p* >= 50%).

### Statistical model

We describe in this section the statistical model developed for this study.

### Notation

We consider an individual *j*. The infection status **I**_**j**_ indicates whether individual *j* was infected by MAYV and/or CHIKV. The ensemble of all possible infection statuses is **I** = {(1,0), (0,1), (0,0), (1,1)} where the first and second element denotes whether an individual was infected by MAYV and CHIKV, respectively. We denote $t_j^{{{\mathrm{M}}}}$ the value of the MAYV RFI for individual *j* ($t_j^{{{\mathrm{C}}}}$ for CHIKV). **θ** is the parameter vector. Individual sociodemographic characteristics are denoted **X**_**j**_.*w*_*j*_ is the sampling weight for individual *j*, indicating the number of people in the population represented by individual *j* in the sample (see details in ref. ^18^). $\varphi (x|\mu ,\sigma )$ is the probability density function of a normal distribution of mean *μ* and standard deviation *σ*.

### Hierarchical structure of the model

We start with the scenario where the infection status is known and then move on to when it is unknown. We can break down the probability of measured RFIs and infection status into two components:

1 $$P\left( {t_j^{{{\mathrm{M}}}},t_j^{{{\mathrm{C}}}},{{{\mathbf{I}}}}_{{{\mathbf{j}}}}|{{{\mathbf{\theta }}}},{{{\mathbf{X}}}}_{{{\mathbf{j}}}}} \right) = P\left( {t_j^{{{\mathrm{M}}}},t_j^{{{\mathrm{C}}}}|{{{\mathbf{I}}}}_{{{\mathbf{j}}}},{{{\mathbf{\theta }}}},{{{\mathbf{X}}}}_{{{\mathbf{j}}}}} \right)P\left( {{{{\mathbf{I}}}}_{{{\mathbf{j}}}}|{{{\mathbf{\theta }}}},{{{\mathbf{X}}}}_{{{\mathbf{j}}}}} \right).$$

The first part represents the antibody dynamics model and the second part the virus circulation model.

### Antibody dynamics model

We model the serological response of an individual as the result of infections by MAYV and/or CHIKV. Several mechanisms contribute to the assay outcome: infection leads to a boost of the RFI for the infecting virus; cross-reactivity may also translate into a rise of the RFI when an individual is infected with another virus. We derive the four probabilities of measured RFIs given the infection status:

1. If an individual has not been infected, the log RFIs are drawn from a normal distribution centered around a background response ($\mu _0^{{{\mathrm{M}}}}$ for MAYV and $\mu _0^{{{\mathrm{C}}}}$ for CHIKV) with standard deviations *ε*^M^ and *ε*^C^:

2 $$P\left( {t_j^{{{\mathrm{M}}}},t_j^{{{\mathrm{C}}}}|{{{\mathbf{I}}}}_{{{\mathbf{j}}}} = (0,0),{{{\mathbf{\theta }}}},{{{\mathbf{X}}}}_{{{\mathbf{j}}}}} \right) = \varphi \left( {t_j^{{{\mathrm{M}}}}|\mu _0^{{{\mathrm{M}}}},\varepsilon ^{{{\mathrm{M}}}}} \right) \times \varphi \left( {t_j^{{{\mathrm{C}}}}|\mu _0^{{{\mathrm{C}}}},\varepsilon ^{{{\mathrm{C}}}}} \right).$$

The standard deviations translate individual variations in the RFI.

2. If an individual has been infected by MAYV only, MAYV log RFI is drawn from a normal distribution of mean $\mu _0^{{{\mathrm{M}}}} + \mu ^{{{\mathrm{M}}}}$ with standard deviation *ε*^M^ Cross-reactivity is modeled as an increase in CHIKV log RFI by a factor that is proportional to MAYV RFI $t_j^{{{\mathrm{M}}}}$ after infection

3 $$\begin{array}{l}P\left( {t_j^{{{\mathrm{M}}}},t_j^{{{\mathrm{C}}}}|{{{\mathbf{I}}}}_{{{\mathbf{j}}}} = (1,0),{{{\mathbf{\theta }}}},{{{\mathbf{X}}}}_{{{\mathbf{j}}}}} \right) = \\ \varphi \left( {t_j^{{{\mathrm{M}}}}|\mu _0^{{{\mathrm{M}}}} + \mu ^{{{\mathrm{M}}}},\varepsilon ^{{{\mathrm{M}}}}} \right) \times \varphi \left( {t_j^C|\mu _0^{{{\mathrm{C}}}} + \mu ^{{{{\mathrm{M}}}} \to {{{\mathrm{C}}}}}t_j^{{{\mathrm{M}}}},\varepsilon ^{{{\mathrm{C}}}}} \right)\end{array}$$

where $\mu ^{{{{\mathrm{M}}}} \to {{{\mathrm{C}}}}}$ is the proportional multiplicative term.

3. If an individual has been infected by CHIKV only, we similarly model the increase in CHIKV RFI and cross-reactivity by

4 $$\begin{array}{l}P\left( {t_j^{{{\mathrm{M}}}},t_j^{{{\mathrm{C}}}}|{{{\mathbf{I}}}}_{{{\mathbf{j}}}} = (0,1),{{{\mathbf{\theta }}}},{{{\mathbf{X}}}}_{{{\mathbf{j}}}}} \right) = \\ \varphi \left( {t_j^{{{\mathrm{M}}}}|\mu _0^M + \mu ^{{{{\mathrm{C}}}} \to {{{\mathrm{M}}}}}t_j^{{{\mathrm{C}}}},\varepsilon ^{{{\mathrm{M}}}}} \right) \times \varphi \left( {t_j^C|\mu _0^{{{\mathrm{C}}}} + \mu ^{{{\mathrm{C}}}},\varepsilon ^{{{\mathrm{C}}}}} \right){{{\mathrm{.}}}}\end{array}$$

4. If an individual has been infected by both MAYV and CHIKV, the response is

5 $$\begin{array}{l}P\left( {t_j^{{{\mathrm{M}}}},t_j^C|{{{\mathbf{I}}}}_{{{\mathbf{j}}}} = (1,1),{{{\mathbf{\theta }}}},{{{\mathbf{X}}}}_{{{\mathbf{j}}}}} \right) = \\ \varphi \left( {t_j^{{{\mathrm{M}}}}|\mu _0^M + \mu ^M,\varepsilon ^M} \right) \times \varphi \left( {t_j^C|\mu _0^{{{\mathrm{C}}}} + \mu ^{{{\mathrm{C}}}},\varepsilon ^{{{\mathrm{C}}}}} \right){{{\mathrm{.}}}}\end{array}$$

We tested alternative antibody models: a model without cross-reactivity, a model where cross-reactivity exists but does not induce a response proportional to the infecting virus response, and a model where infection by both viruses leads to an increase in RFI larger than the one observed after a single infection. This sensitivity analysis is presented in section Model comparison and sensitivity analysis.

### Virus circulation model

The analysis of age-specific seroprevalence profiles is key to estimate historical patterns of viral circulation and the associated risk factors of exposure. Methods known as serocatalytic models are commonly used to reconstruct the annual force of infection (per capita rate at which susceptible individuals are infected on a given year, FOI) from cross-sectional serological surveys. With *λ*_*i*_ the FOI on year *i*, the probability to survive infection during year *i* for an individual susceptible to infection at the start of year *i* is equal to exp(−*λ*_*i*_). On year *S*, the probability that individual *j* of age *a*_*j*_ was ever infected is

6 $$P_j = 1 - {{{\mathrm{exp}}}}\left( { - \mathop {\sum}\limits_{i = 0}^{a_j} {\lambda _{S - i}} } \right)$$

We compare different competing models of virus circulation to explain the observed seroprevalence. In particular, we consider models characterized by constant circulation and by more irregular epidemics.

1. Constant model

In the constant model, the FOI *λ* is the same every year. The contribution to the likelihood of infected individual *j* of age *a*_*j*_ is

7 $$P_j = 1 - {{{\mathrm{exp}}}}\left( { - a_j\lambda } \right).$$

2. Epidemic model

We consider for the epidemic model a single peak in the annual FOI. A Gaussian model was assumed for the epidemic, in which the FOI on year *i* is given by

8 $$\lambda _i = \bar \alpha \exp \left( { - \left( {i - T} \right)^2} \right),$$

where $\bar \alpha = \alpha /\mathop {\sum}\nolimits_i {\exp \left( { - \left( {i - T} \right)^2} \right)}$. This model has two parameters: *T* is the year at which the FOI is maximal, *α* is the total FOI.

### Predictors of infection in the serocatalytic models

In addition to temporal variations of the FOI, we also explore the role of age, housing, income, sex and living in a particular region as predictors of the risk of infection by MAYV and CHIKV. We included these predictors in the model by introducing multiplicative factors to the FOI. The vector of sociodemographic characteristics of individual *j* is

$${{{\mathbf{X}}}}_{{{\mathbf{j}}}} = ({{{{\mathrm{age}}}}_j,{{{\mathrm{regioin}}}}_j,{{{\mathrm{environment}}}}_j,{{{\mathrm{sex}}}}_j,{{{\mathrm{housing}}}}_j,{{{\mathrm{income}}}}_j}).$$

The total cumulative strengths of MAYV and CHIKV infection for individual *j* are

9 $${{\Lambda }}_j^{{{\mathrm{M}}}} = \mathop {\sum }\limits_{i = 0}^{agej} F^{{{\mathrm{M}}}}({{{\mathbf{X}}}}_{{{\mathbf{j}}}},i) \times \lambda _{2017 - i}^{{{\mathrm{M}}}}$$

10 $${{\Lambda }}_j^{{{\mathrm{C}}}} = \mathop {\sum }\limits_{i = 0}^{agej} F^{{{\mathrm{C}}}}\left( {{{{\mathbf{X}}}}_{{{\mathbf{j}}}},i} \right) \times \lambda _{2017 - i}^{{{\mathrm{C}}}}$$

where $\lambda _{2017 - i}^{{{\mathrm{M}}}}$ and $\lambda _{2017 - i}^{{{\mathrm{C}}}}$ are terms characterizing the annual variations of the FOI and the multiplicative factors are given by (for MAYV)

$$\begin{array}{l}F^{{{\mathrm{M}}}}({{{{\boldsymbol{X}}}}_{{{\boldsymbol{j}}}},i}) = f_1^{{{\mathrm{M}}}}({{{{\mathrm{age}}}}_j - i})f_2^{{{\mathrm{M}}}}({{{{\mathrm{region}}}}_j})f_3^{{{\mathrm{M}}}}\\ ({{{{\mathrm{environment}}}}_j})f_4^{{{\mathrm{M}}}}({{{{\mathrm{sex}}}}_j})f_5^{{{\mathrm{M}}}}({{{{\mathrm{housing}}}}_j})f_6^{{{\mathrm{M}}}}({{{{\mathrm{income}}}}_j})\end{array}$$

where $f_1^{{{\mathrm{M}}}}({{{{\mathrm{age}}}}_j - i})$ is the relative susceptibility to MAYV infections of individuals at age age_*j*_−*i* compared with adults (children are defined as under 20 years old). $f_2^{{{\mathrm{M}}}}({{{{\mathrm{region}}}}_j})$,$f_3^{{{\mathrm{M}}}}({{{{\mathrm{environment}}}}_j}),f_4^{{{\mathrm{M}}}}({{{{\mathrm{sex}}}}_j})$,$f_5^{{{\mathrm{M}}}}({{{\mathrm{housing}}}}_j)$,$f_6^{{{\mathrm{M}}}}({{{{\mathrm{income}}}}_j})$ are, respectively, the relative susceptibility of:

– inhabitants of region_*j*_ (High Maroni, Low Maroni, Kourou, Cayenne, High Oyapock, Low Oyapock, Interior) compared with inhabitants of High Maroni
– inhabitants of environment_*j*_ (urban or rural) areas compared with inhabitants of urban areas
– sex_*j*_ (males or females) compared with males
– people living in housing_*j*_ (carbet or other housings) compared with people living in carbets
– people earning income_*j*_ (high or low income) compared to a low income.

The terms $\lambda _{2017 - i}^{{{\mathrm{M}}}}$ and $\lambda _{2017 - i}^{{{\mathrm{C}}}}$ therefore represent the FOI on year 2017-*i* of a male adult, with low income, living in a carbet in urban part of High Maroni.

In the context of the model that considers two circulating viruses and individual specific risks of infection, we extend Eq. (6) and obtain the infection probabilities:

11 $$\begin{array}{l}P\left( {{{{\mathbf{I}}}}_{{{\mathbf{j}}}} = (0,0)|{{{\mathbf{\theta }}}},{{{\mathbf{X}}}}_{{{\mathbf{j}}}}} \right) = {{{\mathrm{e}}}}^{ - {{\Lambda }}_j^{{{\mathrm{M}}}}}{{{\mathrm{e}}}}^{ - {{\Lambda }}_j^{{{\mathrm{C}}}}}\\ P\left( {{{{\mathbf{I}}}}_{{{\mathbf{j}}}} = (1,0)|{{{\mathbf{\theta }}}},{{{\mathbf{X}}}}_{{{\mathbf{j}}}}} \right) = \left(1 - {{{\mathrm{e}}}}^{ - {{\Lambda }}_j^{{{\mathrm{M}}}}}\right){{{\mathrm{e}}}}^{ - {{\Lambda }}_j^{{{\mathrm{C}}}}}\\ P\left( {{{{\mathbf{I}}}}_{{{\mathbf{j}}}} = (0,1)|{{{\mathbf{\theta }}}},{{{\mathbf{X}}}}_{{{\mathbf{j}}}}} \right) = {{{\mathrm{e}}}}^{ - {{\Lambda }}_j^{{{\mathrm{M}}}}} \left(1 - {{{\mathrm{e}}}}^{ - {{\Lambda }}_j^{{{\mathrm{C}}}}}\right)\\ P\left( {{{{\mathbf{I}}}}_{{{\mathbf{j}}}} = (1,1)|{{{\mathbf{\theta }}}},{{{\mathbf{X}}}}_{{{\mathbf{j}}}}} \right) = \left(1 - {{{\mathrm{e}}}}^{ - {{\Lambda }}_j^{{{\mathrm{M}}}}}\right)\left(1 - {{{\mathrm{e}}}}^{ - {{\Lambda }}_j^{{{\mathrm{C}}}}}\right)\end{array}$$

Regional differences in exposures were included as a multiplicative factor in the FOI but we assumed no spatial difference in the timing of epidemics. The CHIKV outbreak started in the region of Kourou and then spread to the rest of the territory, but this is a pattern that cannot be reconstructed from the serological survey. To detect spatial variation of *T*, significant differences in the seroprevalence of infants between the different regions would be required. However, only 15 of them are 2 years old and 9 are 3 years old over the whole territory. Among the children under 5 years old, only 12% (5/43) were found to having been infected by CHIKV. Therefore, we lacked of statistical power to identify the spatial spread of CHIKV.

Inference: In practice the infection status is unknown and only the RFIs are observed. The contribution to the likelihood of individual *j* has to be summed over all possible infection statuses and is

12 $$\begin{array}{l}L_j = P\left( {t_j^{{{\mathrm{M}}}},t_j^{{{\mathrm{C}}}}|{{{\mathbf{\theta }}}},{{{\mathbf{X}}}}_{{{\mathbf{j}}}}} \right) = \\ \mathop {\sum}\limits_{I_k \in I} {P\left( {t_j^{{{\mathrm{M}}}},t_j^{{{\mathrm{C}}}}|{{{\mathbf{I}}}}_{{{\mathbf{j}}}} = {{{\mathbf{I}}}}_{{{\mathbf{k}}}},{{{\mathbf{\theta }}}},{{{\mathbf{X}}}}_{{{\mathbf{j}}}}} \right)P({{{\mathbf{I}}}}_{{{\mathbf{j}}}} = {{{\mathbf{I}}}}_{{{\mathbf{k}}}}|{{{\mathbf{\theta }}}},{{{\mathbf{X}}}}_{{{\mathbf{j}}}})} \end{array}$$

where the first part of the sum was derived in Eqs. (2–5) and the second part in Eq. (11).

### Priors

Flat priors where chosen for most of the antibody model and viral circulation parameters. The prior distributions are Uniform (0,5) for *μ*^M^*,μ* ^C^*, μ*^M→C^*, μ*^C→M^*, ε*^M^*, ε*^C^, the FOI *λ* (constant model), *α* (Epidemic model). The cross-reactive terms were allowed to vary to be either greater or smaller than the terms of the infecting pathogen response. For the Gaussian model of epidemics, the parameter of the peak position *T* is Uniform (1967, 2017). Informative priors were chosen for$\mu _0^{{{\mathrm{M}}}}$ (Normal (0,1)) and $\mu _0^{{{\mathrm{C}}}}$ (Normal (−0.4,1)). For the parameters characterizing the relative risk of infections, *f*_1_ to *f*_6_, a log normal distribution of mean 0 and variance 3 was chosen. This ensures that the groups chosen as reference have no influence on the inferred relative risks (e.g., the prior of the ratio male/female is the same as the prior female/male)^25^.

### Estimation using MCMC

Parameters were estimated using a Markov Chain Monte Carlo (MCMC) algorithm implemented in the rstan package (version 2.19.2)^26^. The No-U-Turn sampler variant of Hamiltonian Monte Carlo was used to update parameters. Four independent chains of 20000 iterations were ran; the first 10000 iterations correspond to a burnin period.

Ninety-five percent credible intervals were defined as the 2.5% and 97.5% percentiles of the posterior distributions. Parameter estimates for the model for antibody dynamics are given in Supplementary Table 5. Models were compared using the deviance information criterion (DIC). A smaller DIC indicates a better fit. Models with DIC under the smallest DIC+5 were considered equally adequate explanations of the data.

### Selection of explanatory variables

Predictors were chosen as follows. We first ran the model without considering the predictors in the force of infection. We used a univariate logistic regression model to predict MAYV and/or CHIKV infections determined by the model with socio-demographic or environmental variables. Variables with an odds ratio significantly different than 1 were added as an explanatory variable in the FOI model.

### Model-based classification of MAYV and CHIKV infections

Conditional on parameter value **θ**, the probability that individual *j* has an infection profile **I**_**j**_ is given by

13 $$P\left( {{{{\mathbf{I}}}}_{{{\mathbf{j}}}}|t_j^{{{\mathrm{M}}}},t_j^{{{\mathrm{C}}}},{{{\mathbf{\uptheta }}}},{{{\mathbf{X}}}}_{{{\mathbf{j}}}}} \right) = \frac{{P\left( {t_j^{{{\mathrm{M}}}},t_j^{{{\mathrm{C}}}}|{{{\mathbf{I}}}}_{{{\mathbf{j}}}},{{{\mathbf{\uptheta }}}},{{{\mathbf{X}}}}_{{{\mathbf{j}}}}} \right)P({{{\mathbf{I}}}}_{{{\mathbf{j}}}}|{{{\mathbf{\uptheta }}}},{{{\mathbf{X}}}}_{{{\mathbf{j}}}})}}{{\mathop {\sum}\limits_{{{{\boldsymbol{I}}}}_{{{\boldsymbol{k}}}} \in I} {P\left( {t^{{{\mathrm{M}}}},t^{{{\mathrm{C}}}}|{{{\mathbf{I}}}}_{{{\mathbf{k}}}}{{{\mathbf{\uptheta }}}},{{{\mathbf{X}}}}_{{{\mathbf{j}}}}} \right)P({{{\mathbf{I}}}}_{{{\mathbf{k}}}}|{{{\mathbf{\uptheta }}}},{{{\mathbf{X}}}}_{{{\mathbf{j}}}}})}}$$

where the infection probabilities are given in Eq. (11) and the denominator is a sum over the four infection profiles. The expected infection status for individual *j* is obtained by averaging the probability (Eq. 13) over the posterior distribution of parameters$p\left( {{{{\mathbf{\uptheta }}}}|t_j^{{{\mathrm{M}}}},t_j^{{{\mathrm{C}}}},{{{\mathbf{X}}}}_{{{\mathbf{j}}}}} \right)$

$$P\left( {{{{\mathbf{I}}}}_{{{\mathbf{j}}}}|t_j^{{{\mathrm{M}}}},t_j^{{{\mathrm{C}}}},{{{\mathbf{X}}}}_{{{\mathbf{j}}}}} \right) = {\int} {P\left( {{{{\mathbf{I}}}}_{{{\mathbf{j}}}}|t_j^{{{\mathrm{M}}}},t_j^{{{\mathrm{C}}}},{{{\mathbf{\uptheta }}}},{{{\mathbf{X}}}}_{{{\mathbf{j}}}}} \right)p\left( {{{{\mathbf{\uptheta }}}}|t_j^{{{\mathrm{M}}}},t_j^{{{\mathrm{C}}}},{{{\mathbf{X}}}}_{{{\mathbf{j}}}}} \right)d{{{\mathbf{\uptheta }}}}}$$

which is obtained in practice by summing over the values of **θ** over the MCMC chain

$$P\left( {{{{\mathbf{I}}}}_{{{\mathbf{j}}}}|t_j^{{{\mathrm{M}}}},t_j^{{{\mathrm{C}}}},{{{\mathbf{X}}}}_{{{\mathbf{j}}}}} \right) = \frac{1}{M}\mathop {\sum }\limits_{s = 1}^M P\left( {{{{\mathbf{I}}}}_{{{\mathbf{j}}}}|t_j^{{{\mathrm{M}}}},t_j^{{{\mathrm{C}}}},{{{\mathbf{\uptheta }}}}_{{{\mathbf{s}}}},{{{\mathbf{X}}}}_{{{\mathbf{j}}}}} \right)$$

where *M* is the chain length and **θ**_s_ the parameter vector with index *s* in the chain.

Infection status of an individual is assessed by taking the most likely expected infection status. We plotted in Fig. 3a the areas of infection profiles fixing infection probabilities $P^{{{\mathrm{M}}}} = 1 - {{{\mathrm{e}}}}^{ - {{\Lambda }}_j^{{{\mathrm{M}}}}} = 0.05$ and $P^{{{\mathrm{C}}}} = 1 - {{{\mathrm{e}}}}^{ - {{\Lambda }}_j^{{{\mathrm{C}}}}} = 0.2$ for all individuals. Taking for example **I**_j _= (1,0), Eq. (13) becomes

$$\begin{array}{l}P\left( {{{{\mathbf{I}}}}_{{{\mathbf{j}}}} = (1,0)|t_j^{{{\mathrm{M}}}},t_j^{{{\mathrm{C}}}},{{{\mathbf{\uptheta }}}},{{{\mathbf{X}}}}_{{{\mathbf{j}}}}} \right) = \\ \frac{{P\left( {t_j^{{{\mathrm{M}}}},t_j^{{{\mathrm{C}}}}|{{{\mathbf{I}}}}_{{{\mathbf{j}}}},{{{\mathbf{\uptheta }}}},{{{\mathbf{X}}}}_{{{\mathbf{j}}}}} \right)P^{{{\mathrm{M}}}}(1 - P^{{{\mathrm{C}}}})}}{{\mathop {\sum }\nolimits_{{{{\mathbf{I}}}}_{{{\mathbf{k}}}} \in I} P\left( {t^{{{\mathrm{M}}}},t^{{{\mathrm{C}}}}|{{{\mathbf{I}}}}_{{{\mathbf{k}}}}{{{\mathbf{\uptheta }}}},{{{\mathbf{X}}}}_{{{\mathbf{j}}}}} \right)P({{{\mathbf{I}}}}_{{{\mathbf{k}}}}|{{{\mathbf{\uptheta }}}},{{{\mathbf{X}}}}_{{{\mathbf{j}}}})}}.\end{array}$$

To evaluate the relative importance of *P*^M^ and *P*^C^ in the determination of the infection profile, we plotted the boundaries for various values of the infection probability (Supplementary Fig. 3). We show that values of *P*^M^ and *P*^C^ have little impact on the likely infection profile which is therefore mostly determined by the RFI values.

### The number of infected individuals

The total number of infections was obtained from the individual model-based predictions of infections weighted by the sampling weights. The 95% confidence intervals of the number of infections were estimated from 10,000 bootstrap resamples where each individual is the resampling unit and where each individual weight was renormalized to keep the total weight unchanged (representing the total number of inhabitants in French Guiana or within a subregion of interest).

The annual probability of MAYV infection was estimated with the formula

$$\frac{{\mathop {\sum }\nolimits_j w_jP_j^{{{\mathrm{M}}}}\left( {{{{\mathrm{annual}}}}\,{{{\mathrm{infection}}}}} \right)}}{{\mathop {\sum }\nolimits_j w_j}}$$

where $P_j^{{{\mathrm{M}}}}\left( {{{{\mathrm{annual}}}}\;{{{\mathrm{infection}}}}} \right) = P_j^{{{{\mathrm{M}}}}_{{{{\mathrm{never}}}}\;{{{\mathrm{infected}}}}}} \times P_j^{{{{\mathrm{M}}}}_{{{{\mathrm{infected}}}}\;{{{\mathrm{this}}}}\;{{{\mathrm{year}}}}}}$ is given by

$$P_j^M\left( {{{{\mathrm{annual}}}}{\kern 1pt} {{{\mathrm{infection}}}}} \right) ={{{\mathrm{e}}}}^{ - {{\Lambda }}_j^{{{\mathrm{M}}}}}\left( {1 - \exp \left( { - F^{{{\mathrm{M}}}}\left( {{{{\mathbf{X}}}}_{{{\mathbf{j}}}}, - 1} \right) \times \lambda _{2017}^{{{\mathrm{M}}}}} \right)} \right)$$

Annual infection probabilities for MAYV are reported in Supplementary Table 11.

### Choice of the threshold value in the simple cutoff classification

The single cutoff classification relies on comparing the RFI with a threshold value, independently of the RFI for the other virus. We plotted the histograms of MAYV (Supplementary Fig. 5a) and CHIKV RFIs (Supplementary Fig. 5b), together with the value of the chosen single threshold cutoff. This value was chosen so as to maximize the sum of the sensitivity and specificity (Supplementary Fig. 5c, d), which were obtained by considering the seroneutralization as the reference diagnostic.

### Analysis with a simple cutoff

To assess the importance of taking into account cross-reactivity in the understanding of the epidemiology of CHIKV and MAYV, we fitted standard serocatalytic models to data, considering seroprevalence based on the simple cutoff instead of the quantitative values of the RFI.

As opposed to the model with cross-reactivity, here, a circulation model in which French Guiana experienced a recent MAYV outbreak was as adequate as a model of constant circulation (DIC = 3599 and DIC = 3600); age (OR: 0.8, 95%CrI: 0.4–1.3) and sex were no longer significant risk factors for MAYV infection (OR: 1.2, 95% CrI: 0.9–1.6).

The reason for these discrepancies between the model-based classification and the simple cutoff is that MAYV and CHIKV have contrasting risk factors for infection: males (respectively, females) are more exposed to MAYV (respectively, CHIKV). Many children and women infected by CHIKV in the urban areas are tested positive for MAYV with the simple cutoff, therefore leading to estimate that MAYV was recent and urban in French Guiana.

### Model adequacy

We assessed model adequacy by simulating 100 surveys in a population with the same characteristics (age, sex…) as in the dataset and with parameters drawn from the posterior distribution.

The simulated surveys could reproduce the observed age profile of the RFI distribution in the different regions except in High Oyapock (Supplementary Fig. 1). For this region, we explored if a model combining constant MAYV circulation along with a MAYV outbreak could improve the fit. While such model improved the fit for MAYV RFIs, it worsened it for CHIKV RFIs (Supplementary Fig. 2), and no overall improvement in terms of DIC was observed (Supplementary Table 6).

In addition, we tested the ability of our approach to identify the correct epidemiological (constant force of infection vs. epidemic transmission), we simulated serological surveys for each of these scenarii. Both surveys had 500 individuals with age randomly drawn between 1 and 70. In the first simulation, we assumed a constant annual probability of infection of 0.01. In the second, we assumed an outbreak that happened in 1980 and infected 20% of the population. We fitted the epidemic and constant model on the two datasets. The DIC shows we are able to identify the correct scenario: we obtained a difference of DIC of 35 for the constant model survey and of 42 for the epidemic model survey. We also estimated the posterior distribution of the parameters. The probability of infection in the first model is 0.94% (95% CrI: 0.78–1.11%) (real value = 1%). For the outbreak model we found *T* to be 1979 (95% CrI: 1977–1981) and the probability of infection is 22% (95% CrI: 16–28%).

### Evaluation of the statistical framework

We also performed a simulation study to evaluate the performance of our statistical framework. One survey was simulated with parameters equal to the posterior mean we estimated in our analysis. We ran our MCMC algorithm on these simulated data. Parameters of antibody model dynamics (Supplementary Table 2) and model-based classification of infections for the number of infected by MAYV and/or CHIKV in the simulation (Supplementary Table 3) were well estimated.

### Model comparison and sensitivity analysis

To ensure that our main conclusions were robust to modeling assumptions, we explored and compared different model variants in a sensitivity analysis. We considered variations of the model presented in this study, by

• Considering other models of antibody response after infection
• Assuming different models of virus circulation
• Removing the predictors from the model of virus circulation
• Different responses for males and females.

### Alternative antibody models

We considered three alternative models for the antibody response to infection. In a first model we assumed that there is no cross-reactivity. Equations (3, 4) of the antibody dynamics model are in this case replaced by:

14 $$\begin{array}{l}P\left( {t_j^{{{\mathrm{M}}}},t_j^{{{\mathrm{C}}}}|{{{\mathbf{I}}}}_{{{\mathbf{j}}}} = (1,0),{{{\mathbf{\theta }}}},{{{\mathbf{X}}}}_{{{\mathbf{j}}}}} \right) = \\ \varphi \Big(t_j^{{{\mathrm{M}}}}{{{\mathrm{|}}}}\mu _0^{{{\mathrm{M}}}} + \mu ^{{{\mathrm{M}}}},\varepsilon ^{{{\mathrm{M}}}} \Big) \times \varphi \left(t_j^{{{\mathrm{C}}}}{{{\mathrm{|}}}}\mu _0^{{{\mathrm{C}}}},\varepsilon ^{{{\mathrm{C}}}}\right)\end{array}$$

15 $$P\left( {t_j^{{{\mathrm{M}}}},t_j^{{{\mathrm{C}}}}|{{{\mathbf{I}}}}_{{{\mathbf{j}}}} = (0,1),{{{\mathbf{\theta }}}},{{{\mathbf{X}}}}_{{{\mathbf{j}}}}} \right) = \varphi \left(t_j^{{{\mathrm{M}}}}{{{\mathrm{|}}}}\mu _0^{{{\mathrm{M}}}},\varepsilon ^{{{\mathrm{M}}}}\right) \times \varphi \left(t_j^{{{\mathrm{C}}}}{{{\mathrm{|}}}}\mu _0^{{{\mathrm{C}}}} + \mu ^{{{\mathrm{C}}}},\varepsilon ^{{{\mathrm{C}}}}\right)$$

In a second model, we included cross-reactivity terms that are not proportional to the response of the other virus, and for which the Eqs. (3) and (4) in the antibody dynamics model are replaced by

16 $$\begin{array}{l}P\left( {t_j^{{{\mathrm{M}}}},t_j^{{{\mathrm{C}}}}|{{{\mathbf{I}}}}_{{{\mathbf{j}}}} = (1,0),{{{\mathbf{\theta }}}},{{{\mathbf{X}}}}_{{{\mathbf{j}}}}} \right) = \\ \varphi \left(t_j^{{{\mathrm{M}}}}{{{\mathrm{|}}}}\mu _0^{{{\mathrm{M}}}} + \mu ^{{{\mathrm{M}}}},\varepsilon ^{{{\mathrm{M}}}}\right) \times \varphi \left(t_j^{{{\mathrm{C}}}}{{{\mathrm{|}}}}\mu _0^{{{\mathrm{C}}}} + \mu ^{{{{\mathrm{M}}}} \to {{{\mathrm{C}}}}},\varepsilon ^{{{\mathrm{C}}}}\right)\end{array}$$

17 $$\begin{array}{l}P\left( {t_j^{{{\mathrm{M}}}},t_j^{{{\mathrm{C}}}}|{{{\mathbf{I}}}}_{{{\mathbf{j}}}} = (0,1),{{{\mathbf{\theta }}}},{{{\mathbf{X}}}}_{{{\mathbf{j}}}}} \right) = \\ \varphi \left(t_j^{{{\mathrm{M}}}}{{{\mathrm{|}}}}\mu _0^{{{\mathrm{M}}}} + \mu ^{{{{\mathrm{C}}}} \to {{{\mathrm{M}}}}}{{{\mathrm{,}}}}\varepsilon ^{{{\mathrm{M}}}}\right) \times \varphi \left(t_j^{{{\mathrm{C}}}}{{{\mathrm{|}}}}\mu _0^{{{\mathrm{C}}}} + \mu ^{{{\mathrm{C}}}},\varepsilon ^{{{\mathrm{C}}}}\right)\end{array}$$

In a third model, we considered that infection by both viruses leads to a boosting of the RFI that includes direct response and cross-reactivity. Equation (5) becomes

18 $$\begin{array}{l}P\left( {t_j^{{{\mathrm{M}}}},t_j^{{{\mathrm{C}}}}|{{{\mathbf{I}}}}_{{{\mathbf{j}}}} = (1,1),{{{\mathbf{\theta }}}},{{{\mathbf{X}}}}_{{{\mathbf{j}}}}} \right) = \\ \varphi \left(t_j^{{{\mathrm{M}}}}{{{\mathrm{|}}}}\mu _0^{{{\mathrm{M}}}} + \mu ^{{{\mathrm{M}}}} + \mu ^{{{{\mathrm{C}}}} \to {{{\mathrm{M}}}}}t_j^{{{\mathrm{C}}}},\varepsilon ^{{{\mathrm{M}}}}\right) \times \varphi \left(t_j^{{{\mathrm{C}}}}{{{\mathrm{|}}}}\mu _0^{{{\mathrm{C}}}} + \mu ^{{{\mathrm{C}}}} + \mu ^{{{{\mathrm{M}}}} \to {{{\mathrm{C}}}}}t_j^{{{\mathrm{M}}}},\varepsilon ^{{{\mathrm{C}}}}\right)\end{array}$$

Values of the DIC show strong support for the model with proportional increase of the response due to cross- reactivity (DIC = 10,343, vs. 11,832, 10,531, and 10,364 for the absence of cross-reactivity, the other cross-reactivity model, and the increased boosting model, respectively).

### Alternative models of virus circulation

We tested four combinations of virus circulation models where (i) MAYV is constant and CHIKV is epidemic, (ii) both MAYV and CHIKV are epidemic, (iii) both MAYV and CHIKV are constant, (iii) MAYV is epidemic and CHIKV circulation is constant. DICs of the different models are shown in Supplementary Table 6. The best fitting model was the one where the FOI was constant for MAYV but epidemic for CHIKV; this scenario was therefore considered as our baseline model.

Given model adequacy (Supplementary Fig. 1), we also considered a model where MAYV circulation in high Oyapock was characterized by both constant circulation and an epidemic (Supplementary Fig. 2). This model improved the fit for MAYV RFIs but gave worse results for CHIKV RFIs. No overall improvement in terms of DIC was observed (Supplementary Table 6).

### Alternative models for risk factors of infection

We fitted the data by removing one predictor of the FOI at a time. DIC indicates higher support for the model with all the predictors (Supplementary Table 7).

### Different responses for males and females

To test the hypothesis that males and females had different boosting, we estimated the model parameters on the subsets of males and females, respectively. We obtained small differences in the model estimates. The MAYV response for females was estimated to be 2.39 (95% CrI: 2.23–2.58) compared to 2.04 (95% CrI: 1.9–2.18) for males. Similarly females had a slightly higher CHIKV response of 3.71 (95% CrI: 3.65–3.75) vs. 3.55 (95% CrI: 3.48–3.62) for males. However we found larger individual variation among males compared to females (parameters *ε*^M^ and *ε*^C^). The mean posterior of *ε*^M^ was 0.51 (95% CrI: 0.50–0.53) for females and 0.54 (95% CrI: 0.52–0.56) for males. The mean posterior of *ε*^C^ was 0.39 (95% CrI: 0.38–0.41) for females and 0.45 (95% CrI: 0.43–0.47) for males (see Supplementary Table 8). Cross-reactivity parameters were estimated to be similar. Overall, the results on prevalence were similar to the baseline where we did not account for differential boosting. The total number of infected individuals was 73 males and 58 females for MAYV (total = 131 compared to 133 with the baseline model) and 192 males and 350 females for CHIKV (total = 542, which is the same as the baseline model).

### Reporting summary

Further information on research design is available in the Nature Research Reporting Summary linked to this article.

## Supplementary information

Supplementary Information

Reporting Summary

Description of Additional Supplementary Files

Supplementary Software 1

## Data Availability

The following data is available in a format that maintains anonymity of survey participants from the GitHub link at https://github.com/nathoze/Mayaro. For each individual: age group (10-year classes), MAYV RFI, CHIKV RFI, region (Maroni, Coast, Interior and High Oyapock), sex, income (high or low), environment (urban or rural), and sampling weight.

## Code availability

Code is available from the GitHub link at https://github.com/nathoze/Mayaro.

## References

[1] Esposito DLA, Fonseca B (2017). Will Mayaro virus be responsible for the next outbreak of an arthropod-borne virus in Brazil?. Braz. J. Infect. Dis..

[2] Hotez PJ, Murray KO (2017). Dengue, West Nile virus, chikungunya, Zika-and now Mayaro. PLoS Negl. Trop. Dis..

[3] Mavian C (2017). Emergence of recombinant Mayaro virus strains from the Amazon basin. Sci. Rep.

[4] Lednicky J (2016). Mayaro virus in child with acute Febrile Illness, Haiti, 2015. Emerg. Infect. Dis..

[5] Paniz-Mondolfi AE, Rodriguez-Morales AJ, Blohm G, Marquez M, Villamil-Gomez WE (2016). ChikDenMaZika syndrome: the challenge of diagnosing arboviral infections in the midst of concurrent epidemics. Ann. Clin. Microbiol. Antimicrob..

[6] Wolfe ND, Daszak P, Kilpatrick AM, Burke DS (2005). Bushmeat hunting, deforestation, and prediction of zoonoses emergence. Emerg. Infect. Dis..

[7] Halsey ES (2013). Mayaro virus infection, Amazon Basin region, Peru, 2010-2013. Emerg. Infect. Dis..

[8] Acosta-Ampudia Y (2018). Mayaro: an emerging viral threat?. Emerg. Microbes Infect..

[9] Brunini S (2017). High frequency of Mayaro virus IgM among febrile patients, Central Brazil. Emerg. Infect. Dis..

[10] PAHO/WHO. 1 May 2019: Mayaro fever-epidemiological alert. https://www.paho.org/hq/index.php?option=com_content&view=article&id=15123:1-may-2019-mayaro-fever-epidemiological-alert&Itemid=42346&lang=en (2019).

[11] Salje H (2019). Nationally-representative serostudy of dengue in Bangladesh allows generalizable disease burden estimates. Elife.

[12] Pezzi L (2019). GloPID-R report on Chikungunya, O’nyong-nyong and Mayaro virus, part I: biological diagnostics. Antiviral Res..

[13] Cellule de l’institut de veille sanitaire en région Antilles-Guyane. Point Epidémiologique de janvier 2015: Le chikungunya dans les Antilles 1–6, (in French, 2015).

[14] Cellule de l’institut de veille sanitaire en région Antilles-Guyane. Point Epidémiologique de juillet 2014: Le chikungunya dans les Antilles 1–7, (in French, 2014).

[15] Fritzell C (2016). Knowledge, attitude and practices of vector-borne disease prevention during the emergence of a new arbovirus: implications for the control of Chikungunya virus in French Guiana. PLoS Negl. Trop. Dis..

[16] Llagonne-Barets M (2016). A case of Mayaro virus infection imported from French Guiana. J. Clin. Virol..

[17] Talarmin A (1998). Mayaro virus fever in French Guiana: isolation, identification, and seroprevalence. Am. J. Trop. Med. Hyg..

[18] Flamand C (2019). Impact of Zika virus emergence in French Guiana: a large general-population seroprevalence survey. J. Infect. Dis..

[19] Aubry M (2018). Seroprevalence of dengue and Chikungunya virus antibodies, French Polynesia, 2014-2015. Emerg. Infect. Dis..

[20] Van Bortel W (2014). Chikungunya outbreak in the Caribbean region, December 2013 to March 2014, and the significance for Europe. Eurosurveillance.

[21] Salje H (2016). How social structures, space, and behaviors shape the spread of infectious diseases using chikungunya as a case study. Proc. Natl Acad. Sci USA.

[22] Epelboin Y (2018). Successes and failures of sixty years of vector control in French Guiana: what is the next step?. Mem. Inst. Oswaldo Cruz.

[23] Fritzell C (2018). Current challenges and implications for dengue, chikungunya and Zika seroprevalence studies worldwide: a scoping review. PLoS Negl. Trop. Dis..

[24] Cao-Lormeau VM (2016). Guillain-Barre Syndrome outbreak associated with Zika virus infection in French Polynesia: a case-control study. Lancet.

[25] Cauchemez S (2009). Household transmission of 2009 pandemic influenza A (H1N1) virus in the United States. N. Engl. J. Med..

[26] Stan Development Team. RStan: the R interface to Stan. R package version 2.19.3. http://mc-stan.org (2020).

