# Author Correction: Reconstructing Mayaro virus circulation in French Guiana shows frequent spillovers

**DOI:** 10.1038/s41467-023-36843-z

**Published:** 2023-02-24

**Authors:** Nathanaël Hozé, Henrik Salje, Dominique Rousset, Camille Fritzell, Jessica Vanhomwegen, Sarah Bailly, Matthieu Najm, Antoine Enfissi, Jean-Claude Manuguerra, Claude Flamand, Simon Cauchemez

**Affiliations:** 1 Mathematical Modelling of Infectious Diseases Unit, Institut Pasteur, UMR2000, CNRS, 75015 Paris, France; 2 Department of Genetics, University of Cambridge, Cambridge, UK; 3 Arbovirus National Reference Center, Institut Pasteur in French Guiana, Cayenne, French Guiana; 4 Epidemiology Unit, Institut Pasteur in French Guiana, Cayenne, French Guiana; 5 Environment and Infectious Risks Unit, Institut Pasteur, Paris, France

**Keywords:** Statistical methods, Viral infection, Epidemiology

Correction to: *Nature Communications* 10.1038/s41467-020-16516-x, published online 05 June 2020

In this article the VEO grant agreement No. 874735 relating to European Union’s Horizon 2020 research and innovation program for S.C. was omitted. The original article has been corrected.

