# Supplementary material for: Reconstructing Mayaro virus circulation in French Guiana shows frequent spillovers
Source: Nat Commun. 2020 Jun 5;11:2842. doi: 10.1038/s41467-020-16516-x (PMC7275077; doi:10.1038/s41467-020-16516-x)
Supplement: Supplementary file 1 — Supplementary Information [file 41467_2020_16516_MOESM1_ESM.pdf]

Supplementary Information: Reconstructing Mayaro virus circulation in French Guiana shows frequent spillovers

Nathanaël Hozé, Henrik Salje, Dominique Rousset, Camille Fritzell, Jessica Vanhomwegen, Sarah Bailly, Matthieu Najm, Antoine Enfissi, Jean-Claude Manuguerra, Claude Flamand, Simon Cauchemez

This file contains Supplementary Figures 1-7 and Supplementary Tables 1-11.

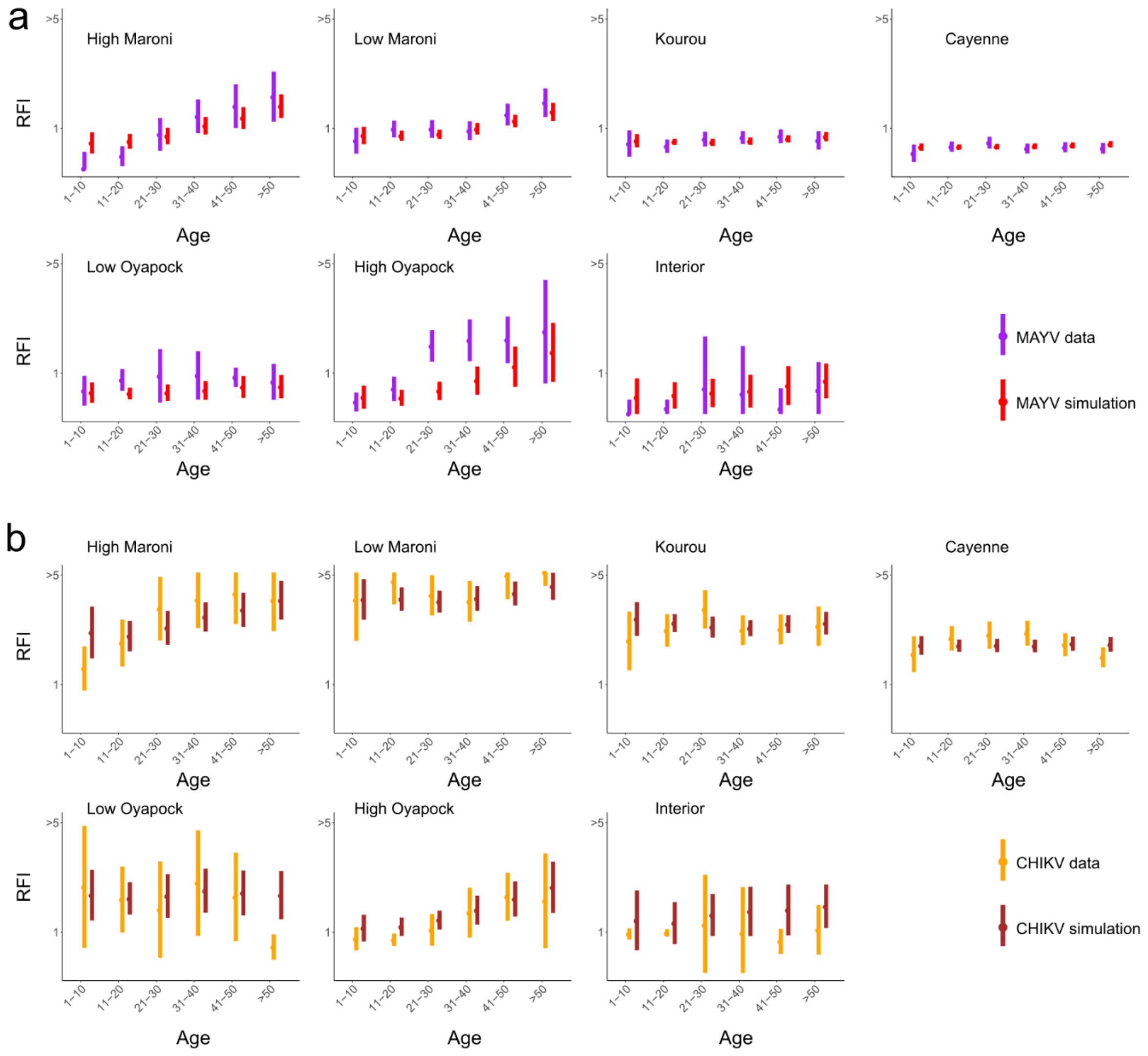

Supplementary Figure 1: Model adequacy. Observed and expected age profile of the mean RFI in the different regions, where expected RFI distributions were obtained from 100 simulations using parameters drawn from the posterior distribution. Bars represent the standard error of the mean for the observations and the standard deviation of the average RFI for the simulations. **a**, MAYV; **b**, CHIKV.

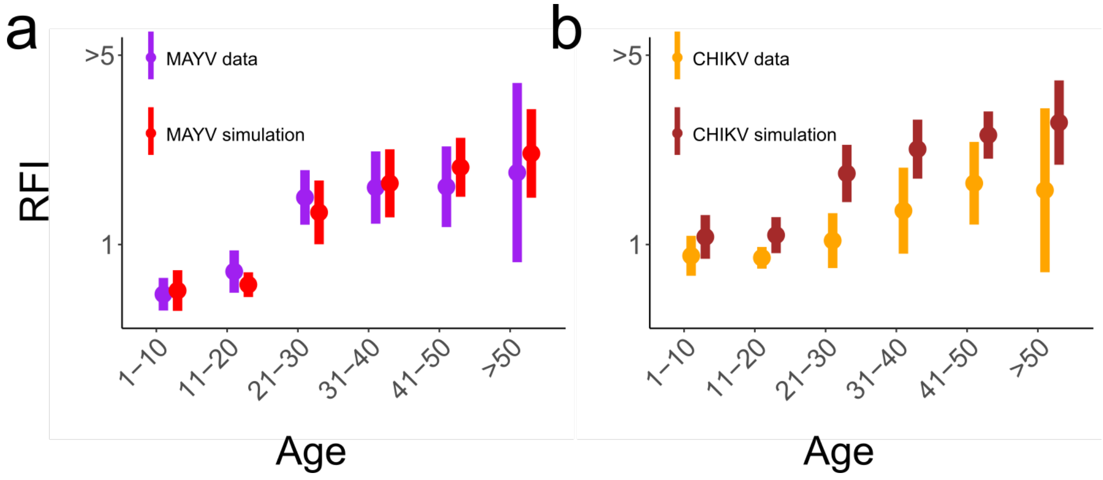

Supplementary Figure 2: Model adequacy. Observed and expected age profile of the mean RFI in the High Oyapock region, assuming a combined model of constant circulation and epidemic for MAYV. The expected RFI distributions were obtained from 100 simulations using parameters drawn from their posterior distribution. Bars represent the standard error of the mean. **a**, MAYV; **b**, CHIKV.

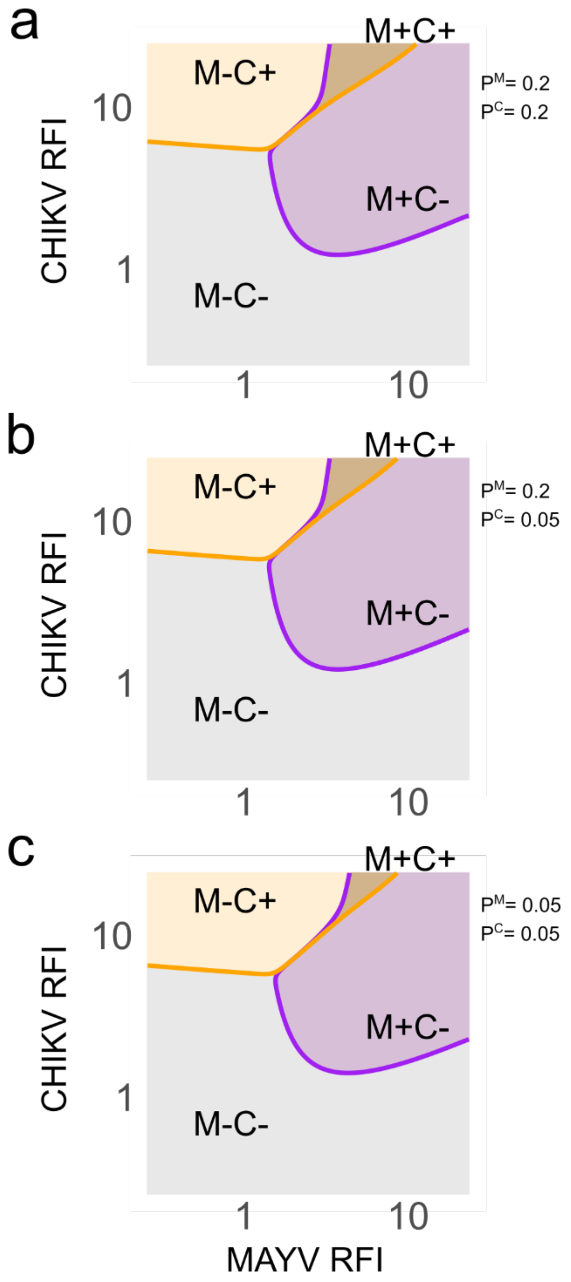

Supplementary Figure 3: Influence of the assumed infection probabilities on the model-based classification. Regions of infection profile are determined by the probability of being infected given the RFIs and the population infection probability. Different probabilities of infection by MAYV and CHIKV (P^M and P^C) were tested. Colors indicate the most likely infection profile and solid lines are the boundaries of equal probability between profiles.

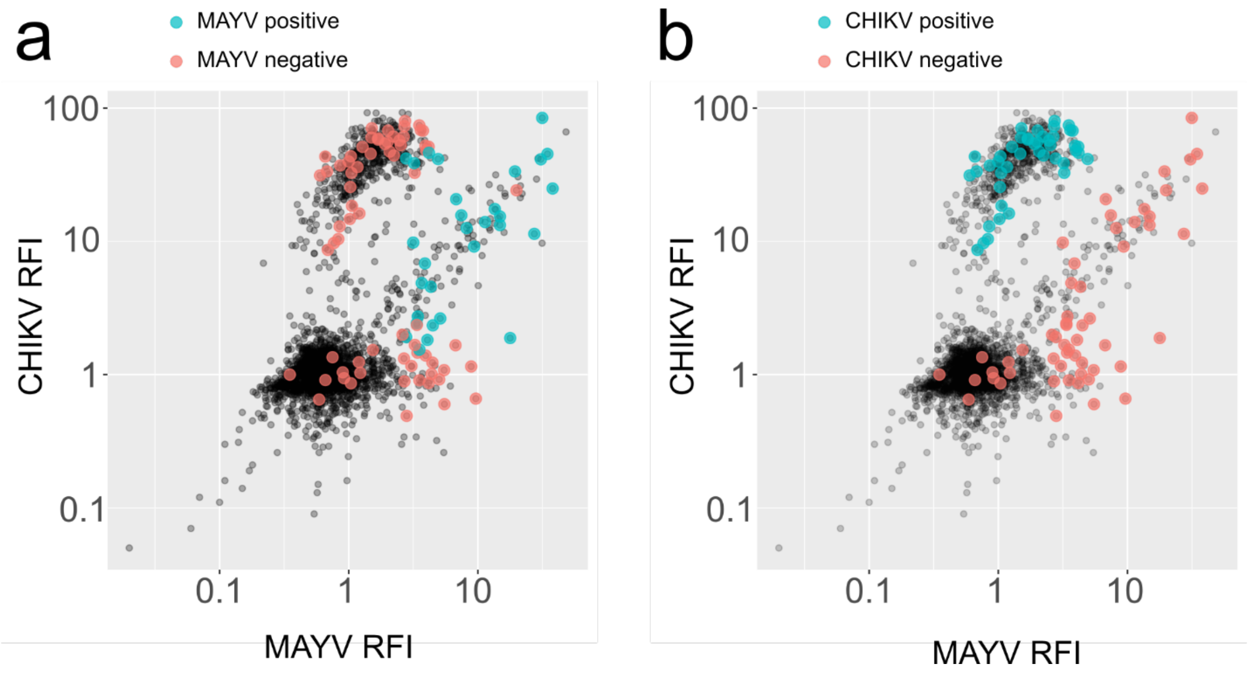

Supplementary Figure 4: Assessment of the model-based classification with a seroneutralization assay. We plotted the RFI values for the 2,697 samples in black and used colors for the 100 samples that were chosen for additional testing with seroneutralization. Blue indicates a positive result for seroneutralization for MAYV (**a**) and CHIKV (**b**) and red a negative result.

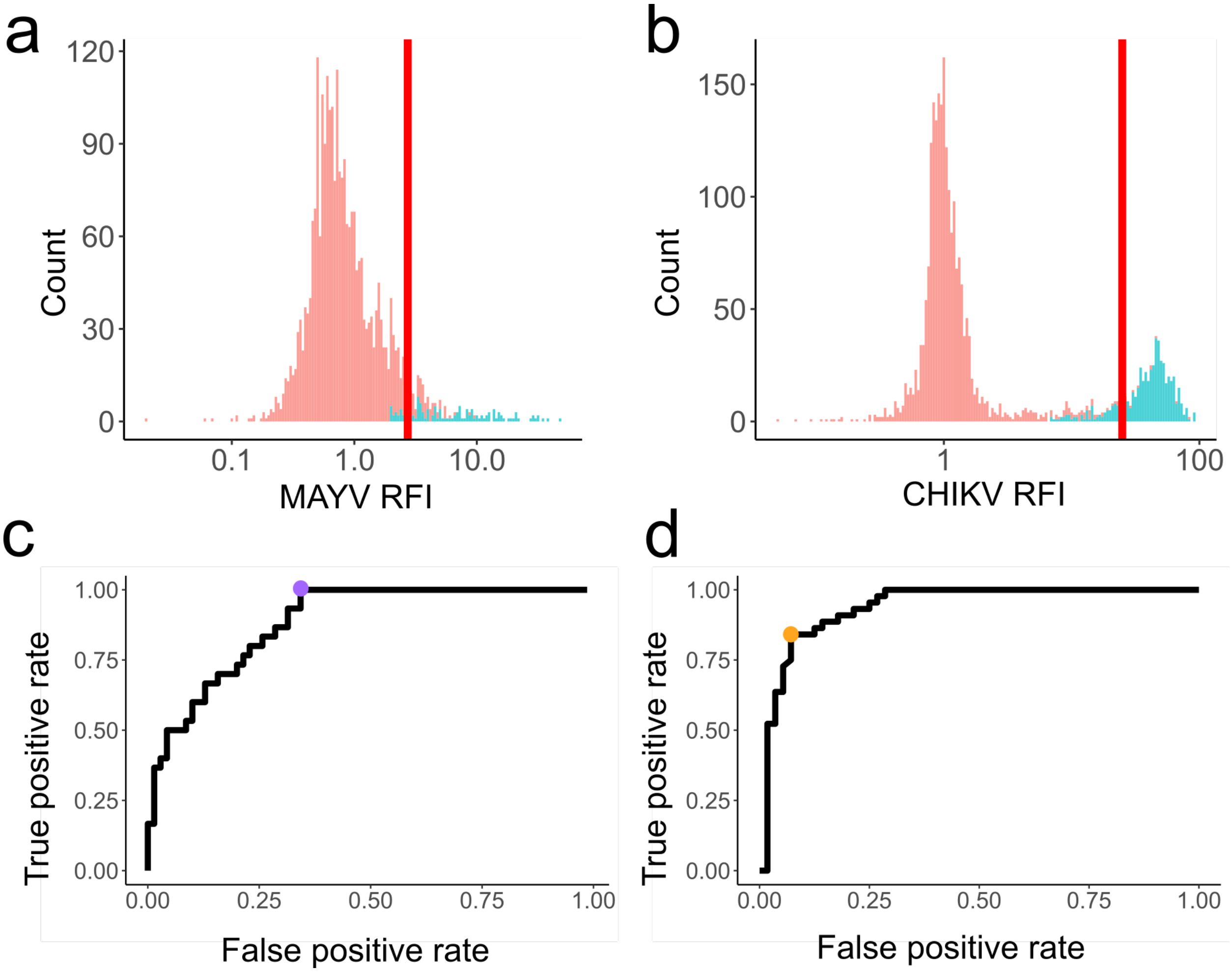

Supplementary Figure 5: Choice of a RFI threshold for seropositivity in the simple cutoff model. a,b, Histograms of the RFI for MAYV (**a**) and CHIKV (**b**) (n=2,697). The red vertical line corresponds to the chosen threshold value (2.7 for MAYV and 24.9 for CHIKV). Blue bars represent the positive cases according to the model-based classification, and pink bars the negative cases. **c, d**, ROC curves of the classification of MAYV (**c**) and CHIKV (**d**) with the single cutoff, taking the seroneutralization as the reference. The purple (**c**) and orange (**d**) points show where the true positive rate and false positive rate stand for the chosen threshold value.

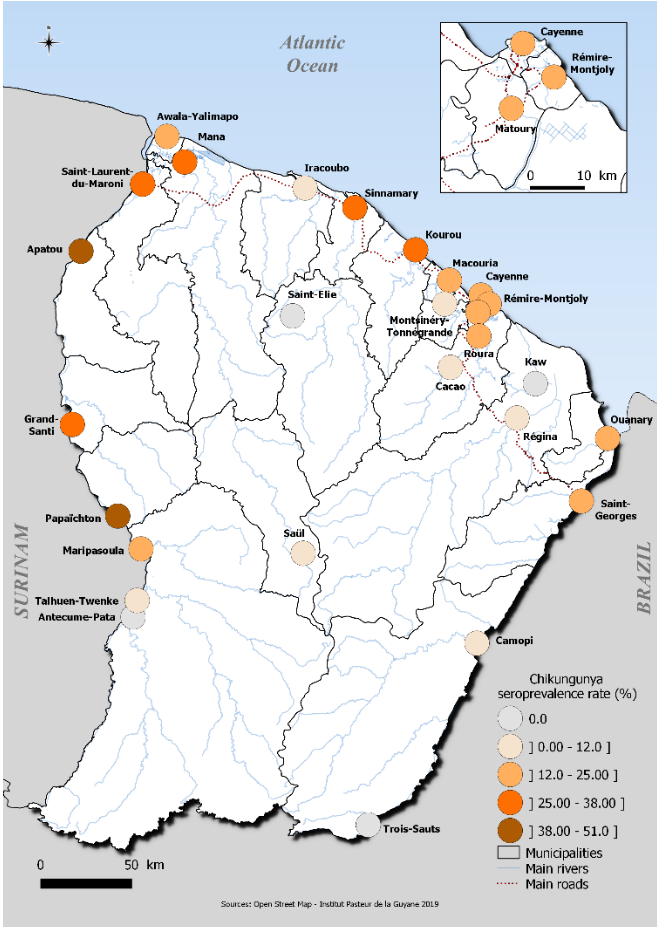

Supplementary Figure 6. Map of CHIKV seroprevalence in each municipality.

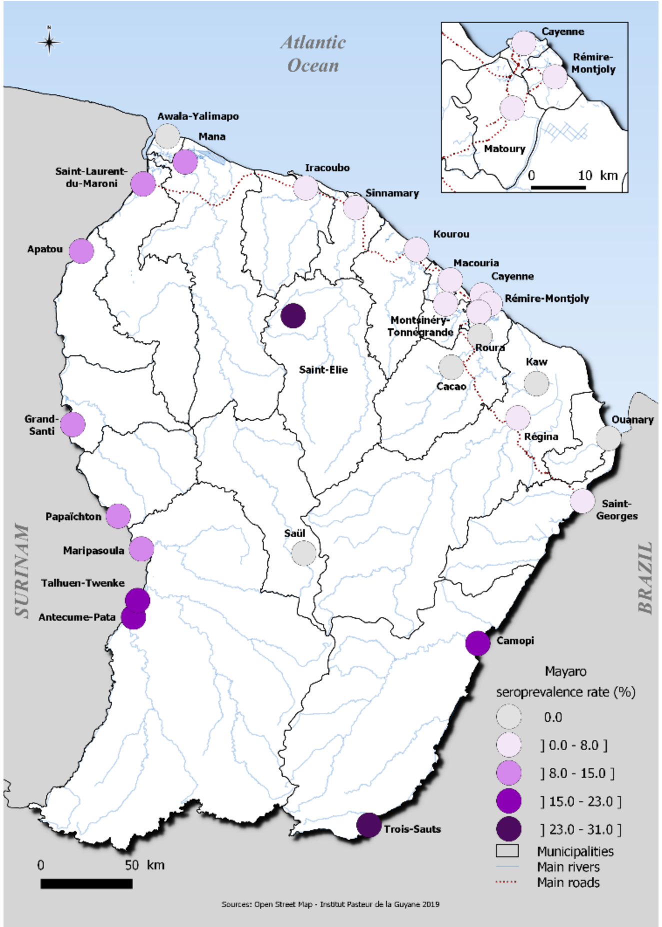

Supplementary Figure 7. Map of MAYV seroprevalence in each municipality.

Supplementary Table 1: Number of individuals included in the survey for the seven different regions.

Region	Municipalities	N
High Maroni	Papaïchton, Maripasoula, Antecume-Pata, Talhuen-Twenke	194
Low Maroni	Grand-Santi, Apatou, Saint-Laurent du Maroni	424
Kourou	Awala, Iracoubo, Mana, Kourou, Macouria, Sinnamary	699
Cayenne	Cayenne, Regina, Remire, Matoury, Montsinnery, Roura, Cacao, Kaw	1121
Low Oyapock	Saint-Georges, Ouanary	99
High Oyapock	Camopi, Trois-Sauts	115
Interior	Saint-Elie, Saül	45

Supplementary Table 2: Validation of the statistical framework to estimate antibody model parameters. Input column corresponds to parameter values used in the simulation study. We report the mean and 95 % credible intervals of parameters estimated from the simulated dataset.

**Parameter**	**Input**	**Estimate (95% CrI)**
μ_0^{M}	-0.40	-0.4 (-0.41 – -0.38)
μ_0^{C}	-0.01	0 (-0.01 – 0.02)
μ^{M}	2.19	2.19 (2.1 – 2.28)
μ^{C}	3.64	3.61 (3.57 – 3.64)
$\mu^{\mathrm{C}\rightarrow\mathrm{M}}$	0.22	0.21 (0.2 – 0.22)
$\mu^{\mathrm{M}\rightarrow\mathrm{C}}$	1.05	1.06 (1.02 – 1.11)
ε^{M}	0.52	0.53 (0.51 – 0.54)
ε^{C}	0.42	0.42 (0.41 – 0.44)

Supplementary Table 3: Validation of the statistical framework to estimate the infection status. The table compares the true number of infections in the simulated dataset to the one estimated from this dataset.

**Infections**	**Simulations Input**	**Inference estimates (mean and 95% CrI)**
MAYV+	130	128 (126 - 131)
CHIKV+	574	572 (572 – 574)
MAYV- and CHIKV-	2017	2016 (2014 – 2018)
MAYV+ and CHIKV-	106	108 (106 – 110)
MAYV- and CHIKV+	550	553 (550 – 554)
MAYV+ and CHIKV+	24	20 (18 – 23)

Supplementary Table 4: Sample sizes for each age class in the different regions of French Guiana.

Age class	High Maroni	Low Maroni	Kourou	Cayenne	Low Oyapock	High Oyapock	Interior
1-10	23	52	70	134	23	18	6
11-20	34	95	132	179	21	15	3
21-30	36	72	95	149	14	26	8
31-40	37	73	152	177	14	24	3
41-50	32	68	99	159	15	23	13
>50	32	64	151	323	12	9	12

Supplementary Table 5: Mean and 95 % credible intervals of the parameters of the model of antibody dynamics.

Parameter	Description	Estimate (95% CrI)
μ_0^{M}	Baseline MAYV RFI	-0.40 (-0.43 – -0.38)
μ_0^{C}	Baseline CHIKV RFI	-0.02 (-0.04 – 0)
μ^{M}	Increase of MAYV RFI after MAYV infection	2.2 (2.08 – 2.31)
μ^{C}	Increase of CHIKV RFI after CHIKV infection	3.64 (3.60 – 3.68)
$\mu^{\mathrm{C}\rightarrow\mathrm{M}}$	Increase of MAYV RFI after CHIKV infection	0.22 (0.21 – 0.23)
$\mu^{\mathrm{M}\rightarrow\mathrm{C}}$	Increase of CHIKV RFI after MAYV infection	1.05 (1.01 – 1.1)
ε^{M}	Standard deviation of MAYV RFI	0.52 (0.51 – 0.54)
ε^{C}	Standard deviation of CHIKV RFI	0.42 (0.41 – 0.43)

Supplementary Table 6: Comparison of the baseline model with different models of circulation.

Model	DIC
MAYV constant, CHIKV outbreak (baseline)	10343
MAYV and CHIKV outbreaks	10380
MAYV and CHIKV constant	10379
MAYV outbreak, CHIKV constant	10394
MAYV constant and outbreak in High Oyapock, CHIKV constant	10337

Supplementary Table 7: Comparison of the baseline model with models where one of the predictors is discarded in the force of infection.

Model	DIC
Baseline model	10343
Remove age	10349
Remove housing	11938
Remove income	10371
Remove region	10457
Remove environment	10373
Remove sex	10352

Supplementary Table 8: Mean and 95% credible intervals of the parameters of the model of antibody dynamics when considering only males and only females.

Parameter	Males only (mean and 95% CrI) (n=1108)	Females only (mean and 95% CrI) (n=1589)
μ_0^{M}	-0.42 [-0.45, -0.38]	-0.39 [-0.42, -0.36]
μ_0^{C}	-0.01 [-0.04, 0.01]	-0.03 [-0.05, 0]
μ^{M}	2.04 [1.9, 2.18]	2.39 [2.23, 2.58]
μ^{C}	3.55 [3.48, 3.62]	3.71 [3.65, 3.75]
$\mu^{\mathrm{C}\to\mathrm{M}}$	0.2 [0.18, 0.22]	0.22 [0.21, 0.24]
$\mu^{\mathrm{M}\to\mathrm{C}}$	1.06 [0.99, 1.13]	1.04 [0.97, 1.11]
ε^{M}	0.54 [0.52, 0.56]	0.51 [0.5, 0.53]
ε^{C}	0.45 [0.43, 0.47]	0.39 [0.38, 0.41]

Supplementary Table 9: Estimated number of infected individuals in French Guiana. Mean numbers are obtained by summing the weights of individuals of the survey infected according to the model. 95% confidence intervals are obtained by bootstrap resampling where individuals are the resampling units (10,000 resamples).

**Region**	**MAYV infected**	**CHIKV infected**
High Maroni	1150 (732 – 1612)	2391 (1755 – 3048)
Low Maroni	3468 (2664 – 4958)	13215 (11350 – 15143)
Kourou	807 (357 – 1351)	10364 (8670 – 12111)
Cayenne	1063 (431 – 1823)	25744 (21960 – 29746)
Low Oyapock	45 (0 – 144)	768 (380 – 1215)
High Oyapock	320 (214 – 437)	15 (0 – 47)
Interior	109 (0 – 219)	6 (0 – 20)
Total	7263 (5764 – 8868)	52503 (47845 – 57290)

Supplementary Table 10: Estimated seroprevalence in the 27 communities. Mean seroprevalence were obtained by averaging the individual model-based classification weighted by the corresponding sampling weights. 95% confidence intervals were obtained with 10,000 bootstrap resampled data.

Municipality	MAYV infected (%)	CHIKV infected (%)
Antecume-Pata	16.68 (4.07 – 31.06)	0 (0 – 0)
Apatou	9.89 (3.05 – 18)	44.3 (31.5– 57.3)
Awala	0 (0 – 0)	16.02 (7.04 – 26.31)
Cacao	0 (0 – 0)	3.39 (0 – 11.18)
Camopi	18.16 (10.4 – 26.9)	1.42 (0 – 4.4)
Cayenne	0.32 (0 – 0.81)	18.06 (14.44 – 21.82)
Grand-Santi	10.55 (3.57 – 19.18)	31.5 (20.0 – 44.0)
Iracoubo	4.62 (0 – 11.64)	4.73 (0 – 12.49)
Kaw	0 (0 – 0)	0 (0 – 0)
Kourou	1.88 (0.5 – 3.6)	28.2 (22.89 – 33.69)
Macouria	0.76 (0 – 2.37)	14.25 (9.08 – 19.99)
Mana	9.24 (3.5 – 16.01)	33 (23.39 – 42.93)
Maripasoula	11.53 (4.8 – 19.36)	21.09 (11.39 – 31.35)
Matoury	1.55 (0.28 – 3.1)	16.21 (11.49 – 21.2)
Montsinnery	0.88 (0 – 2.86)	8.65 (2.88 – 15.82)
Ouanary	0 (0 – 0)	23.26 (0 – 49.1)
Papaichton	8.51 (1.53 – 17.14)	50.3 (35.36 – 65.9)
Regina	6.09 (1.08 – 12.24)	4.3 (0.95 – 9.05)
Remire	0.65 (0 – 2.02)	16.15 (10.93 – 21.68)
Roura	0 (0 – 0)	13.27 (2.89 – 25.82)
Saint-Elie	30.55 (0 – 59.42)	0 (0 – 0)

Saint-Georges	0.76 (0 – 2.41)	12.1 (5.58 – 19.68)
Saint-Laurent	9.2 (6.0 – 12.8)	31.95 (26.43 – 37.74)
Saul	0 (0 – 0)	1.85 (0 – 6.07)
Sinnamary	3.64 (0 – 11.4)	25.02 (11.32 – 40.33)
Talhuen-Twenke	16.17 (6.25 – 29.16)	7.22 (0 – 18.34)
Trois-Sauts	29.1 (14.5 – 46.4)	0 (0 – 0)

Supplementary Table 11: Mean and 95% credible intervals of MAYV annual probability of infection and annual number of infections.

Region	Infection probability (in %)	Number of infections
High Maroni	0.54 (0.38 – 0.72)	52 (36 – 68)
Low Maroni	0.43 (0.32 – 0.55)	172 (130 – 216)
Kourou	0.097 (0.057 – 0.14)	46 (27 – 69)
Cayenne	0.04 (0.019 – 0.072)	61 (29 – 112)
Low Oyapock	0.08 (0.0098 – 0.21)	4.9 (0.69 – 13)
High Oyapock	0.97 (0.71 – 1.2)	14 (11 – 18)
Interior	0.38 (0.11 – 0.75)	2.5 (0.75 – 5)
Total	0.14 (0.11 – 0.17)	351 (287 – 427)
