# Supplementary material for: Reconstructing Mayaro virus circulation in French Guiana shows frequent spillovers
Source: Nat Commun. 2020 Jun 5;11:2842. doi: 10.1038/s41467-020-16516-x (PMC7275077; doi:10.1038/s41467-020-16516-x)
Supplement: Supplementary file 2 — Reporting Summary [file 41467_2020_16516_MOESM2_ESM.pdf]

nature research

Corresponding author(s): Simon Cauchemez

Last updated by author(s): Apr 23, 2020

Reporting Summary

Nature Research wishes to improve the reproducibility of the work that we publish. This form provides structure for consistency and transparency in reporting. For further information on Nature Research policies, see Authors & Referees and the Editorial Policy Checklist .

Statistics

For all statistical analyses, confirm that the following items are present in the figure legend, table legend, main text, or Methods section.

n/a	Confirmed	
☐	☒	The exact sample size (*n*) for each experimental group/condition, given as a discrete number and unit of measurement
☐	☒	A statement on whether measurements were taken from distinct samples or whether the same sample was measured repeatedly
☒	☐	The statistical test(s) used AND whether they are one- or two-sided<br>*Only common tests should be described solely by name; describe more complex techniques in the Methods section.*
☐	☒	A description of all covariates tested
☒	☐	A description of any assumptions or corrections, such as tests of normality and adjustment for multiple comparisons
☐	☒	A full description of the statistical parameters including central tendency (e.g. means) or other basic estimates (e.g. regression coefficient) AND variation (e.g. standard deviation) or associated estimates of uncertainty (e.g. confidence intervals)
☒	☐	For null hypothesis testing, the test statistic (e.g. *F*, *t*, *r*) with confidence intervals, effect sizes, degrees of freedom and *P* value noted<br>*Give P values as exact values whenever suitable.*
☐	☒	For Bayesian analysis, information on the choice of priors and Markov chain Monte Carlo settings
☐	☒	For hierarchical and complex designs, identification of the appropriate level for tests and full reporting of outcomes
☒	☐	Estimates of effect sizes (e.g. Cohen's *d*, Pearson's *r*), indicating how they were calculated

Our web collection on statistics for biologists contains articles on many of the points above.

Software and code

Policy information about availability of computer code

Data collection	No software was used for data collection.
Data analysis	Custom code was developed in R (version 3.3.2). MCMC algorithm was performed using the R package rstan (version 2.19.2). The code was uploaded to a public repository https://github.com/nathoze/Mayaro

For manuscripts utilizing custom algorithms or software that are central to the research but not yet described in published literature, software must be made available to editors/reviewers. We strongly encourage code deposition in a community repository (e.g. GitHub). See the Nature Research guidelines for submitting code & software for further information.

Data

Policy information about availability of data

All manuscripts must include a data availability statement. This statement should provide the following information, where applicable:
- Accession codes, unique identifiers, or web links for publicly available datasets
- A list of figures that have associated raw data
- A description of any restrictions on data availability

The following data is available in a format that maintains anonymity of survey participants. For each individual: age group (10-year classes), MAYV RFI, CHIKV RFI, region (Maroni, Coast, Interior and High Oyapock), sex, income (high or low), environment (urban or rural), and sampling weight. The data was uploaded to a public repository https://github.com/nathoze/Mayaro

Field-specific reporting

Please select the one below that is the best fit for your research. If you are not sure, read the appropriate sections before making your selection.

Behavioural & social sciences study design

All studies must disclose on these points even when the disclosure is negative.

Study description	Cross-sectional population-based serological survey and household interviews conducted in French Guiana.
Research sample	French Guiana population.
Sampling strategy	We estimated the sample size for this survey at 2,500 persons distributed in the French Guiana territory based on a 50% seroprevalence, 95% confidence, 90% power and a cluster effect. To reach the desired sample size, a total of 1,600 households were randomly selected for possible participation in the study from household databases maintained by the Geographic information and knowledge dissemination unit of the Regional environment, planning and housing agency and the National Institute of Economic and Statistical Information (INSEE). A stratified simple random sampling was adopted to select households allowing an over-representation of the isolated and small municipalities. The global sampling fraction of the households was 1:49 varying from 1:103 to 1:5 according to the municipality.<br>We employ the following notation to describe the study design:<br>- i: one of the 22 strata (municipalities);<br>- Mi: number of primary sampling units (households) in the ith stratum, i=1, ..., 22;<br>- Si: number of primary sampling units (households) selected from the ith stratum, i=1, ..., 22;<br>- mi: number of primary sampling units (households) actually enrolled in the study from the ith stratum, i=1, ..., 22;<br>- Pi: number of individuals living within the ith stratum, i=1, ..., 22 (census data);<br>- pi: number of individuals actually enrolled in the study from the ith stratum, i=1, ..., 22;<br>We considered that, in each municipality i, the probability of selecting a particular subject was equal to the probability of selecting his household and was (mi/Mi), corresponding to a statistical weight equal to (1/ mi/Mi) = (Mi/mi). This statistical weight indicates the number of people in the population represented by each subject in the sample.<br>We applied a post-stratification adjustment to each of these weights to arrive at the final statistical weight for each subject. This adjustment helped us to weight the age-sex groups within each municipality to match the distribution in the French Guiana total population. Ten age groups ([2-5 years [, [5-10[, [10-15[, [15-20[, [20-25[, [25-35[, [35-45[, [45-55[, [55-65[, and ≥65 years) were defined within male and female groups, and for each age-sex subgroup, we applied an adjustment factor cijk to obtain a final statistical weight wijk = (Mi/mi)*cijk, where i, j, k are the indices of municipalities, sex groups, and age groups, respectively
Data collection	Data were collected through a standardized questionnaire<br>installed on tablets to register demographics, socioeconomics, and household characteristics. A venous blood sample of 10 mL<br>was collected from each participant, in accordance with current biosafety standards. The researcher was not blind to sampling procedure.
Timing	between June and October 2017.
Data exclusions	No data exclusion
Non-participation	No participants dropped out;
Randomization	Participants were not allocated into experimental groups

Reporting for specific materials, systems and methods

We require information from authors about some types of materials, experimental systems and methods used in many studies. Here, indicate whether each material, system or method listed is relevant to your study. If you are not sure if a list item applies to your research, read the appropriate section before selecting a response.

Materials & experimental systems

n/a	Involved in the study	
☐	☒	Antibodies
☐	☒	Eukaryotic cell lines
☒	☐	Palaeontology
☒	☐	Animals and other organisms
☐	☒	Human research participants
☒	☐	Clinical data

Methods

n/a	Involved in the study	
☒	☐	ChIP-seq
☒	☐	Flow cytometry
☒	☐	MRI-based neuroimaging

Antibodies

Antibodies used	Jackson ImmunoResearch Biotin Goat IgG antiHuman IgG Fc - P/N : 109-065-008<br>Supplier : Interchim, P/N : 109-065-008, item 561340<br>lot number : 128570

Validation	NA (secondary antibody)

Eukaryotic cell lines

Policy information about cell lines

Cell line source(s)	Vero cell line : (cells from kidney of african green monkey) ATCC number CCL-81, lot 70005907
Authentication	Validated and certified by ATCC
Mycoplasma contamination	The cell line was not tested for mycoplasma contamination (but used soon after reception from ATCC)
Commonly misidentified lines (See ICLAC register)	no commonly misidentified cell lines were used in the study

Human research participants

Policy information about studies involving human research participants

Population characteristics	French Guiana Population. Sex, age, housing type and location, household structure, socio-economic status.
Recruitment	1600 households were randomly selected. All details are given in Flamand, C. et al., Impact of Zika virus emergence in French Guiana: a large general-population seroprevalence survey. J Infect Dis., 2019 and in the Methods section of the present paper
Ethics oversight	The study was recorded on Clinicaltrials.gov (ID: NCT03210363) and approved by the "Sud-Ouest & Outre-Mer IV" Ethical Research Committee (No.CPP17-007a/2017-A00514-49) and by the French Data Protection Authority (No.DR-2017-324) responsible for ethical issues and protection of individual data collection.

Note that full information on the approval of the study protocol must also be provided in the manuscript.
