# Supplementary material for: Reconstructing Mayaro virus circulation in French Guiana shows frequent spillovers
Source: Nat Commun. 2020 Jun 5;11:2842. doi: 10.1038/s41467-020-16516-x (PMC7275077; doi:10.1038/s41467-020-16516-x)
Supplement: Supplementary file 3 — Description of Additional Supplementary Files [file 41467_2020_16516_MOESM3_ESM.pdf]

Description of Additional Supplementary Files

File Name: Supplementary Software 1
Description: The Supplementary Software contains scripts and data to reproduce key results of the paper. For reasons related to the anonymity of the survey participants, the data were aggregated. For each individual, we provide the age group (in 10-year classes), MAYV RFI, CHIKV RFI, region (Maroni, Coast, Interior and High Oyapock), sex, and sampling weight. Additionally, the seroneutralization results on 100 individuals are provided. The R scripts allow to load the data, plot the data, run the MCMC algorithm, and analyse the parameter estimates. The code was written in R (version 3.3.2) and was tested on a Windows computer. It requires the package rstan.
