# Supplementary material for: Reconstructing Mayaro virus circulation in French Guiana shows frequent spillovers
Source: Nat Commun. 2020 Jun 5;11:2842. doi: 10.1038/s41467-020-16516-x (PMC7275077; doi:10.1038/s41467-020-16516-x)
Supplement: Supplementary file 4 — Supplementary Software 1 [file 41467_2020_16516_MOESM4_ESM.zip › CodeAndData/ReadMe.docx]

**R scripts and Data for “Reconstructing Mayaro virus circulation in French Guiana shows frequent spillovers”**

**Data**

For reasons related to the anonymity of the survey participants, we could not submit the raw data used in the analysis. The following data are available in a format that maintains anonymity of survey participants. For each individual: age group (10-year classes), MAYV RFI, CHIKV RFI, region (Maroni, Coast, Interior and High Oyapock), sex, and sampling weight (file: CHIKVMAYV.csv). Additionally, the seroneutralization results on 100 individuals are provided (file: SeroNeutralisation.csv).

**Code**

The code was written in R (version 3.3.2) and was tested on a Windows computer. It requires the package *rstan*. To use *rstan,* Windows users must previously install Rtools.

The R scripts allow to load the data, plot the data (figure 1), run the MCMC algorithm, and analyse the parameter estimates. The data included here are not the ones described in the manuscript and in particular do not include specific environment, housing and income characteristics which would have made the survey participants identifiable. We allow the user to analyze a modified version of the data where a single category for each of the above characteristic is considered, and where the age of the participant is drawn uniformly within their age class. This data structure cannot be used to reproduce exactly the results, but the main results are similar.

All the scripts can be run sequentially using the script *startHere.R*
